# Growth deregulation and interaction with host hemocytes contribute to tumor progression in a Drosophila brain tumor model

**DOI:** 10.1073/pnas.2221601120

**Published:** 2023-08-07

**Authors:** Chrysanthi Voutyraki, Alexandros Choromidis, Anastasia Meligkounaki, Nikolaos Andreas Vlachopoulos, Vasiliki Theodorou, Sofia Grammenoudi, Emmanouil Athanasiadis, Sara Monticelli, Angela Giangrande, Christos Delidakis, Evanthia Zacharioudaki

**Affiliations:** ^a^Institute of Molecular Biology & Biotechnology, Foundation for Research & Technology Hellas, 70013 Heraklion, Crete, Greece; ^b^Department of Biology, University of Crete, 70013 Heraklion, Crete, Greece; ^c^Institute for Fundamental Biomedical Research, Biomedical Sciences Research Center Alexander Fleming, 16672 Athens, Greece; ^d^Greek Genome Centre, Biomedical Research Foundation of the Academy of Athens, 11527 Athens, Greece; ^e^Medical Image and Signal Processing Laboratory, Department of Biomedical Engineering, University of West Attica, 12243 Athens, Greece; ^f^Institut de Génétique et de Biologie Moléculaire et Cellulaire, 67400 Strasbourg, France; ^g^Centre National de la Recherche Scientifique, UMR7104 Strasbourg, France; ^h^Institut National de la Santé et de la Recherche Médicale, U1258 Strasbourg, France; ^i^Université de Strasbourg, 67404 Strasbourg, France

**Keywords:** neural stem cells, Notch, Drosophila allografts, brain tumor, macrophages

## Abstract

Tumors constantly interact with their microenvironment. Here, we present data on a Notch-induced neural stem cell (NSC) tumor in Drosophila, which can be immortalized by serial transplantation in adult hosts. This tumor arises in the larva by virtue of the ability of Notch to suppress early differentiation–promoting factors in NSC progeny. Guided by transcriptome data, we have addressed both tumor-intrinsic and microenvironment-specific factors and how they contribute to tumor growth and host demise. The growth promoting factors Myc, Imp, and Insulin receptor in the tumor cells are important for tumor expansion and killing of the host. From the host’s side, hemocytes, professional phagocytic blood cells, are found associated with tumor cells. Phagocytic receptors, like NimC1, are needed in hemocytes to enable them to capture and engulf tumor cells, restricting their growth. In addition to their protective role, hemocytes may also increase the host’s morbidity by their propensity to produce damaging extracellular reactive oxygen species.

## Significance

How tumor-intrinsic factors and microenvironment shape brain tumor progression remains an open question. Here, we present a transcriptomic, cellular, and genetic dissection of a neural tumor generated by Notch hyperactivation in Drosophila. We have focused on the differences between the larval primary tumor and its more aggressive version, which emerges soon after transplantation to adults. This has provided insights on tumor growth strategies, like the involvement of Myc, Imp, and the insulin receptor pathway. We found that host macrophages profusely infiltrate the allografted tumor and impede its growth through phagocytosis. Surprisingly, cytokines (TNF, Jak/STAT) that often mediate macrophage-epithelial tumor signaling were not activated in this brain tumor. Our findings contribute to a better understanding of tumorigenesis strategies and tumor–microenvironment interactions.

It is widely appreciated that cell-intrinsic mechanisms are critical in cancer initiation. Often a single genetic insult disrupting oncogenic or tumor suppressor pathways predisposes a cell to cancer formation. Therapies targeting these insults have been highly effective for a few cancers (e.g., leukemias) (1) but not for neurological solid tumors, which remain among the most lethal child and adult cancers. Brain tumors are characterized by cellular diversity; they are born and maintained by “stem cell-like” subpopulations that self-renew and generate progeny by hijacking the same developmental programs that normal Neural Stem Cells (NSCs) use to generate diverse cells types during development and upon tissue repair (2). How brain tumors progress to a malignant state is often the result of an intricate interplay between deregulated developmental programs in cancer NSCs and interactions with the tumor microenvironment (TME) (3). For instance, glioblastoma multiforme (GBM) with high density of macrophage infiltration usually correlates with poor patient prognosis (3, 4). Tumor-associated macrophages (TAMs) either eliminate cancer cells through proinflammatory responses (M1-type macrophages), or sustain tumor growth through anti-inflammatory responses that nurture cancer stem cells, stimulate angiogenesis, suppress adaptive immunity, and ultimately promote metastasis (M2-type macrophages). Many cancer therapeutic strategies combine TAM-targeting agents with chemotherapy and immunotherapy to achieve a synergistic antitumor effect (3).

Genetic insults in various proliferative tissues in Drosophila have been reported to generate malignant tumors (5–7). Recently, researchers have used such fly cancer models to address tumor–macrophage interactions (8). A simple innate immune system grants Drosophila protection from pathogens and parasites. Blood cells, or hemocytes, are a central pillar of the fly immune response. The most abundant hemocyte type, plasmatocytes, are professional phagocytic cells. Like vertebrate macrophages, they are capable of phagocytosing microbes, cell debris, and foreign bodies, and promoting humoral immune responses in other tissues by secreting proinflammatory cytokines (9). A few recent reports show that hemocytes, similar to mammalian TAMs, are attracted to premalignant epithelia (e.g., imaginal disks with *scrib, dlg1, l*(2)*gl* mutations). Whether the immune response initiated by these hemocytes kills the tumor cells or enables their invasion and metastasis (or leaves the tumor unaffected) depends on the precise genetic constitution and physical characteristics of the tumor (10–13).

Whereas epithelial tumors have become a popular cancer model in Drosophila, nonepithelial cancers like nervous system tumors have received less attention. Drosophila larval neural stem cells [or neuroblasts (NBs)] generate most cells of the central nervous system (CNS) and serve as a simple system to study stem cell–derived tumorigenesis. NBs, like mammalian NSCs, divide asymmetrically throughout embryo and larval life to generate neuronal and glial progenitors with limited mitotic potential. Upon each NB division, cytoplasmic asymmetries ensure the inheritance of prodifferentiation factors to the basal, more committed neural progenitor. These factors help switch off the stem-cell program (14) and their perturbation (15–19) can lead to tumor-like NB hyperplasias in the larval CNS. These hyperplasias exhibit uncontrolled proliferation after transplantation to adult hosts. Serial transplantation is a standard way to assay malignancy in Drosophila (20), since larvae live only 5 d and the NB hyperplasias prohibit progression to later stages, due to CNS malformation. It is not known, however, whether these transplanted NSC-like tumors interact with the host’s hemocytes, like epithelial-derived tumors.

We have shown that overactivating Notch signaling in larval NSC lineages (by overexpression of *NΔecd*, a constitutively active form of Notch), results in NSC hyperplasias (21–23). Our genetic and transcriptomic analysis revealed that stemness [e.g., *dpn, E(spl)mγ, wor, klu*] and growth (e.g., *Myc*) transcription factors are up-regulated and turn on the stem cell program in NSC progeny at the expense of differentiation. We have recently shown that these NSC hyperplasias can progress to malignancy upon transplantation (24, 25).

Here, we have gone on to serially allograft this *NΔecd* tumor, in order to follow it at various stages of progression (primary larval tumor, early allografts, or late allografts) and observe how it changes over time. RNA sequencing (RNAseq) and histological analysis revealed that allografted *NΔecd*-tumors shut down neural tissue identity and up-regulate processes related to stress, metabolism and immunity. Growth related genes are important mediators of malignancy. Furthermore, Notch malignant tumors recruit a large number of host hemocytes. These hemocytes have a tumor-suppressive role. Phagoreceptors of the Nimrod family (e.g., *NimC1*) are important for hemocyte–tumor association and their loss leads to impaired capture and phagocytosis of tumor cells, thus enabling the tumor to accelerate its harmful effects.

## Results

### *NΔecd* Allograft Tumors Have Little Differentiated Progeny.

**Fig. 1.**
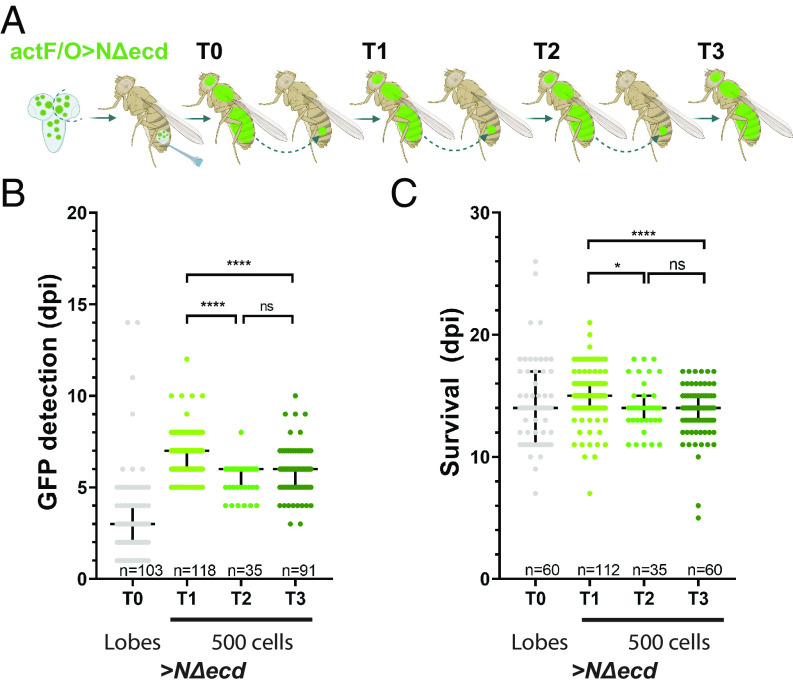
Notch‐induced allograft tumors become more aggressive upon serial transplantations. (*A*) The transplantation assay. Hyperplastic *Act-F/O*>*GFP +ΝΔecd* brain lobes were allografted into the abdomen of adult *w^1118^* females (3 to 4 d after eclosion) (allograft T0). After several days, the tumor was retransplanted into new hosts (allograft T1). This process was repeated twice, up to allograft T3. (*B* and *C*) Scatter plots depicting the distribution of the first day that GFP was mesoscopically detected in hosts (*B*) and the host lifespan (*C*) as days postinjection (dpi). For T0: Whole-brain lobes and T1 to T3: 500 T_(n-1)_-dissociated cells were injected into each host. Middle black lines: median values; low and upper black lines: first to third interquartile ranges (IQRs); *adjusted P (*P*_*adj*_) < 0.05; ****P_adj_ < 0.0001, ns: not significant (ordinary one-way ANOVA, Tukey’s multiple comparisons test). n = the total sample size scored from 3 biologically independent experiments.

We sought to answer whether Notch-induced NSC tumors become more aggressive upon serial transplantation and how their transcriptomic profile is morphed over time. We either exploited the *actin FLP‐out* (*act>STOP>Gal4 or act‐F/O* in brief) system or a *Gal80^ts^-* temporally controlled NSC‐specific *grhNB-Gal4* driver (hereafter called *grh^ts^-Gal4*) to overexpress *NΔecd*, either in random Green Fluorescent Protein (GFP) marked clones (*act-F/O*) or broadly in most larval NSC lineages (*grh^ts^-Gal4*). We thus generated larval CNS hyperplasias (primary tumors) characterized by the presence of ectopic Dpn-positive NSC-like cells at the expense of progenitors and young neurons (Pros-positive cells; *SI Appendix*, Fig. S1*A*). We subsequently transplanted individual *NΔecd* hyperplastic brain lobes into the abdomens of healthy adult female *w^1118^* hosts (transplant T0). The allograft tumors grew in the host flies for approximately 6 to 10 d. Before killing their host, they were removed and some portion was retransplanted into new healthy flies. This process was repeated several times (up to 3 retransplantations; T1 to T3, see Fig. 1*A*). These allograft tumors were mainly composed of NSC-like cells that express Dpn (*SI Appendix*, Fig. S1*A*) and E(spl)mγ (25) and carried few differentiated cells, which were further reduced as the allografts became more advanced (see Pros-positive cells in T2 and T3 in *SI Appendix*, Fig. S1 *A* and *B*).

In order to accurately compare the harmful effects of early (T1) vs. more advanced allografts (T2, T3), we injected a defined number of cancer cells per host. As a pilot, gradual increase in the number of *grh^ts^>NΔecd* T0 tumor cells injected per host fly (from 100 to 1,000 cells, *SI Appendix*, Fig. S1*C*) revealed that the lethality of T1 allograft tumors was proportional to the number of T0 cancer NSC-like cells injected (*SI Appendix*, Fig. S1 *D* and *E*). Even 100 tumor cells reduced the lifespan of the host more than simple sterile injury [Phosphate Buffer Saline (PBS)] (*SI Appendix*, Fig. S1 *F* and *G*). We proceeded to inject 500 cancer cells per host from gradually more progressed tumor stages (T0, T1, T2). We could mesoscopically detect a faster emergence of GFP signal in hosts carrying the more advanced allografts (T2, T3) (Fig. 1*B*) and these hosts died a few days earlier (Fig. 1*C*). Taken together, the faster appearance of GFP, the faster demise of the hosts and the lower number of differentiating cells (*SI Appendix*, Fig. S1 *A* and *B*) suggest that *NΔecd* NSC tumors become somewhat more aggressive upon serial transplantations.

### Transcriptomic Analysis of *NΔecd* Tumors.

We performed expression profiling of cancer cells at various stages of the tumor. For the primary tumor, we hand-dissected larval brains carrying *act‐F/O> NΔecd+GFP* clones, and used Fluorescence-Activated Cell Sorting (FACS) to isolate the GFP-positive population. Whole-tumor masses hand-dissected from the first (T0) and fourth (T3) allograft stages were used as the early and more advanced tumor material, respectively.

**Fig. 2.**
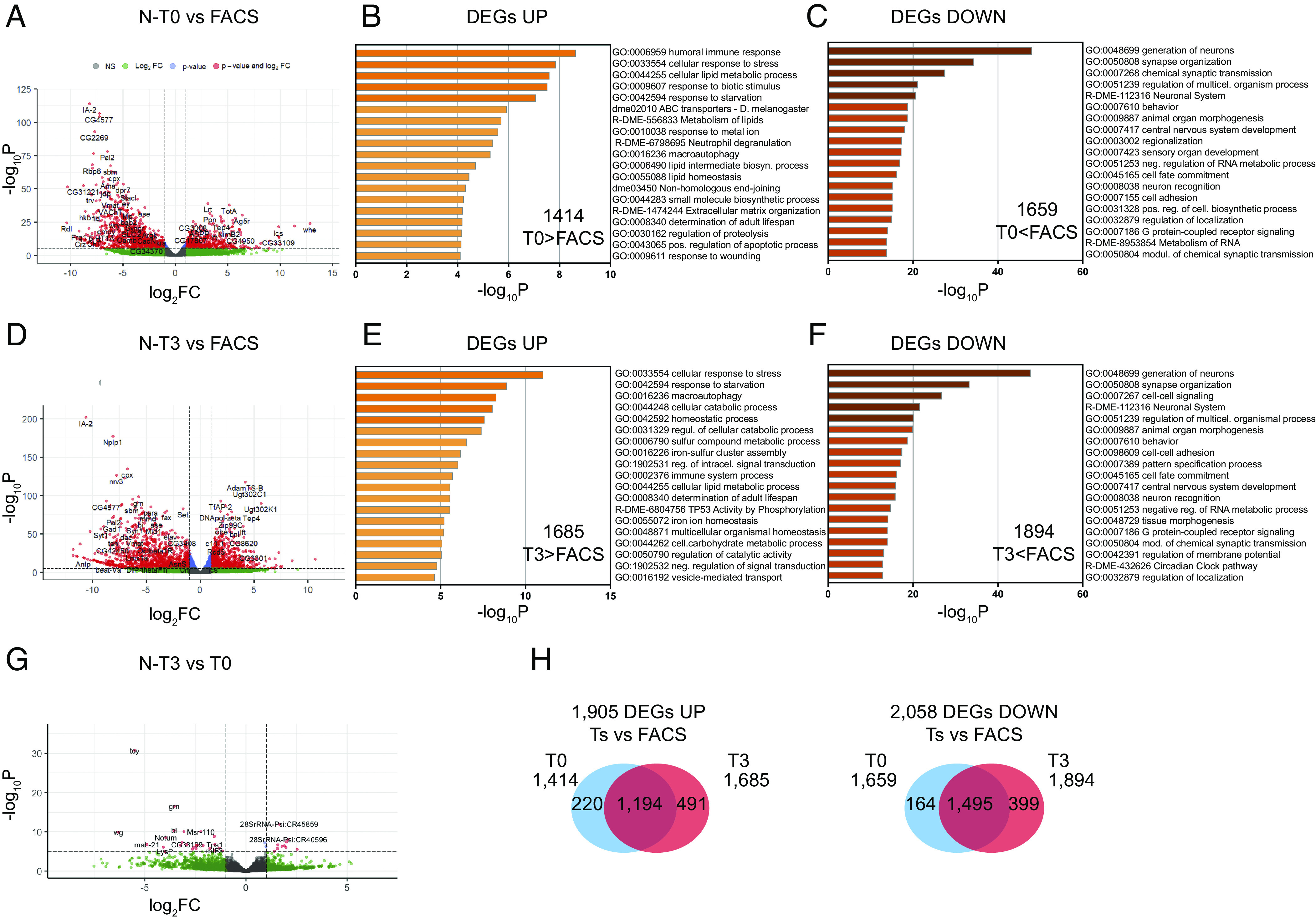
Transcriptomic analysis of *NΔecd* tumors. (*A*, *D*, and *G*) Volcano plots of RNA-seq analysis of all genes in pairwise comparisons of various stages of *NΔecd* tumors: T0 vs. primary (FACS) (*A*), T3 vs. primary (*D*), T3 vs. T0 (*G*). Genes with False Discovery Rate (FDR) ≤ 0.05 and |log_2_FC| ≥ 0.5 are marked in red. (*B*, *C*, *E*, and *F*) Bar graphs of the top enriched GO terms across gene lists of up-regulated (*B* and *E*) and down-regulated (*C* and *F*) genes. (*H*) Venn diagrams of up-regulated (*Left*) and down-regulated (*Right*) genes, in T0 vs. primary and T3 vs. primary comparisons.

Comparing the T0/T3 tumors to the primary lesion (abbreviated as “FACS”), we found 3,963 differentially expressed genes (|logFC| ≥ 0.5 and *P_adj_* ≤ 0.05), 68% of which were common in both T0 and T3 stages (Fig. 2 *A*, *D*, and *H* and Dataset S1). This enormous change, corresponding to almost half of the appreciably expressed genes across samples, is in part due to the elimination of neurons and glia from the allografted samples, but their partial retention in the primary tumor. We have shown earlier that *NΔecd* overexpression causes neuron dedifferentiation with partial penetrance, which decreases with increasing maturation of the affected cell (21, 24). In contrast, comparison of T0 with T3 gave only 107 differentially expressed genes (70 down/ 37 up at the T3 stage; Fig. 2*G* and Dataset S1). These data suggest that the allograft tumor transcriptome remains rather stable over the examined time period (see also correlation heatmap with dendrogram in *SI Appendix*, Fig. S2*A*).

Differentially expressed genes from *NΔecd* T0 and T3 stages (compared to *NΔecd*-FACS) were mined for GO-term enrichment (Fig. 2 *B*, *C*, *E*, and *F* and Dataset S2). Consistent with the elimination of neurons after transplantation, the down-regulated genes were enriched in processes related to brain development and function [e.g., CNS development (GO:0007417), generation of neurons (GO:0048699), synapse organization (GO:0050808), behavior (GO:0007610), etc.] On the other hand, processes related to response to stress (GO: 0033554) and wounding (GO:0009611), starvation (GO:0042594), metabolism [cellular lipid metabolic process (GO:0044255), cellular carbohydrate metabolic process (GO:0044262)], and immunity [e.g. humoral immune response (GO:0006959) and immune system process (GO:0002376)] were enriched in the up-regulated gene sets. The smaller differentially expressed gene sets between T0 and T3 showed no significant enrichment.

The initial defect in the *NΔecd* tumor seems to be the ability of Notch to induce the expression of repressors of neural and glial differentiation in the young progeny cells of NB lineages. The Hes factors Dpn and E(spl)mγ are central among these repressors. Forced expression of *dpn* and *E(spl)mγ*, causes similar larval NB lineage hyperplasias that become malignant (24); we refer to these as *DM* tumors. Similar to *NΔecd* tumors, transcriptome analysis of allografted *DM* tumors had also revealed a loss of differentiation coupled with a shift in metabolism and increased stress (25). In order to address any differences in the transcriptome of *NΔecd* vs. *DM* tumors, we directly compared them to each other at the primary, T0 and T3 stages. Using our criterion of |logFC| ≥ 0.5 and *P_adj_* ≤ 0.05, we found 393, 304, and 469 differentially expressed genes in the primary, T0 and T3 stage, respectively (*SI Appendix*, Fig. S2 *B*, *E*, and *H* and Dataset S3). Enrichment analysis showed that *DM* tumors consistently display higher expression of early neuronal genes (like *erm, pros, hbn, scro, ham, cas, dati*) (*SI Appendix*, Fig. S2 *D*, *G*, and *J* and Dataset S4), all the way to the T3 stage, suggesting a greater degree of differentiation. Genes expressed more highly in *NΔecd* vs. *DM* showed no remarkable enrichment in any processes, other than drug metabolism and sulfur metabolism, both due to higher levels of the antioxidant Glutathione-S-transferase (Gst) enzymes (*SI Appendix*, Fig. S2 *C*, *F*, and *I* and Dataset S4). Other than these small differences, *NΔecd* and *DM* tumors are highly similar at the transcriptome level. In contrast, the transcriptional signature of *NΔecd* tumors is quite distinct from that of normal NBs (26) or of allograft NB tumors originating from defects in asymmetric cell division factors (27) (*SI Appendix*, Fig. S3).

### Genes Related to Growth Are Necessary for Notch-Induced Hyperplasias to Progress to Malignancy.

**Fig. 3.**
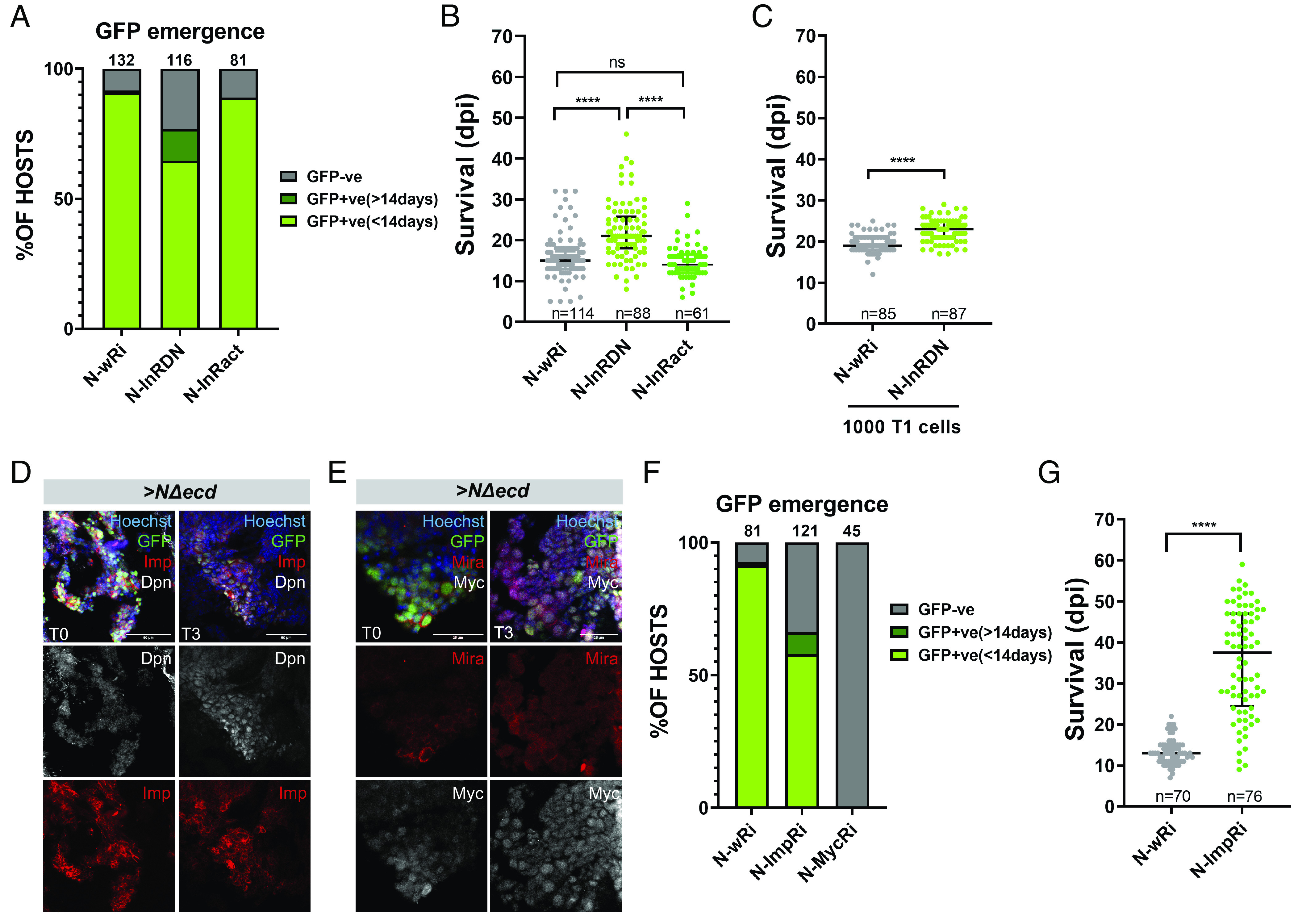
InR, Imp and Myc mediate *NΔecd* allograft tumor growth. (*A* and *F*) Diagrams depicting the proportion of hosts that develop GFP-positive tumors within 14 or >14 d after transplantation of a brain lobe of the indicated genotypes: >*NΔecd* in *w-RNAi* (control) vs. *InR^DN^* or *InR^act^* background (*A*) and >*NΔecd* in *w-RNAi* (control) vs. *Imp-RNAi* or *Myc-RNAi* background (*F*). The total number of allografted flies scored from 3 to 5 independent biological replicates was pooled and is shown above the bars. (*B*, *C*, and *G*) Scatter plots showing host lifespan as dpi of brain lobes (*B* and *G*) or 1,000 T1 cells (*C*) of the indicated genotypes. Since *NΔecd*; *InR^DN^* allografts are growth compromised, we used 1,000 (instead of 500) cells for retransplantation experiments to offer them an advantage. Only GFP-positive hosts were scored; n = the total sample size pooled from 3 to 5 independent biological replicates. Middle black lines: median values; lower and upper black lines: first to third IQRs; *P_adj_ or *P* < 0.05; **** *P*_adj_ or *P* < 0.0001, ns: not significant [ordinary one-way ANOVA, Tukey’s multiple comparisons test for *B*; unpaired *t* test for (*C* and *G*). (*D* and *E*) Confocal images depicting the expression of Imp (red; *D*) or Myc (gray; *E*) in *actinF/O>NΔecd, GFP* T0 or T3 allograft tumor fragments. Hoechst marks nuclei whereas Dpn (gray; *D*) or Mira (red; *E*) marks NSC-like cells. (Scale bar, 50 μm.)

We asked whether growth-related pathways contribute to *NΔecd* induced malignancy. The insulin receptor (InR) pathway is central in coordinating growth with nutrient uptake. InR is expressed, but is not differentially regulated in the allograft *NΔecd* T3 vs. T0 tumors and we tested its role by expressing a dominant negative (*InR^DN^*) or a constitutively active form (*InR^act^*) in *NΔecd* background. Expression of *InR^DN^* mildly reduced the magnitude of *NΔecd*-induced NSC hyperplasia in the larval CNS (*SI Appendix*, Fig. S4 *A* and *B*) and, upon transplantation, it gave rise to allograft tumors less frequently, GFP signal was detected with a delay and hosts survived longer (Fig. 3 *A* and *B*). Even at the T2 stage, *NΔecd*; *InR^DN^* tumors continued to show a delay in killing their hosts (Fig. 3*C*). The converse manipulation, overexpression of *InR^act^*, enlarged the primary hyperplasia (*SI Appendix*, Fig. S4 *A* and *B*) but had no effect on allograft tumor growth (Fig. 3 *A* and *B*). Thus, insulin signaling interacts with active Notch to promote primary and allograft tumor growth at early and progressed stages.

We next examined the role of the growth-regulating TF Myc and the RNA-binding protein Imp, which binds and stabilizes *Myc* mRNA in NBs (28). Both are highly expressed in tumors and are up-regulated as the tumors progress to T3. We confirmed expression of Imp and Myc in T0 and T3 tumors by immunofluorescence (Fig. 3 *D* and *E*). RNAi knockdown of either *Ιmp* or *Myc* (*SI Appendix*, Fig. S4 *E* and *F*) reduced larval NB hyperplasia (*SI Appendix*, Fig. S4 *C* and *D*), but did not altogether abolish the dedifferentiation effect caused by *NΔecd*. Upon allografting, depletion of *Imp* led to a reduced number of hosts developing tumors, a delay in the emergence of GFP-positive tumors and a striking extension in the hosts’ lifespan (Fig. 3 *F* and *G*). Interestingly, these *NΔecd*; *ImpRNAi* tumors did not spread throughout the host (*SI Appendix*, Fig. S4*G*) and were so small that it was impossible to collect enough tumor cells to proceed to T1 stage and beyond. Depletion of *Myc* had an even more dramatic effect in *ΝΔecd* allograft tumor growth, as none of the host flies ever developed detectable T0 tumor (Fig. 3*F*). Therefore, although Imp or Myc depletion has moderate effects at the primary tumor stage (*SI Appendix*, Fig. S4*D*), it dramatically handicaps or abolishes the growth of allografts (Fig. 3 *F* and *G*).

### Hemocytes Are Attracted to *NΔecd* Allograft Tumors but Not to the Primary Hyperplasias.

**Fig. 4.**
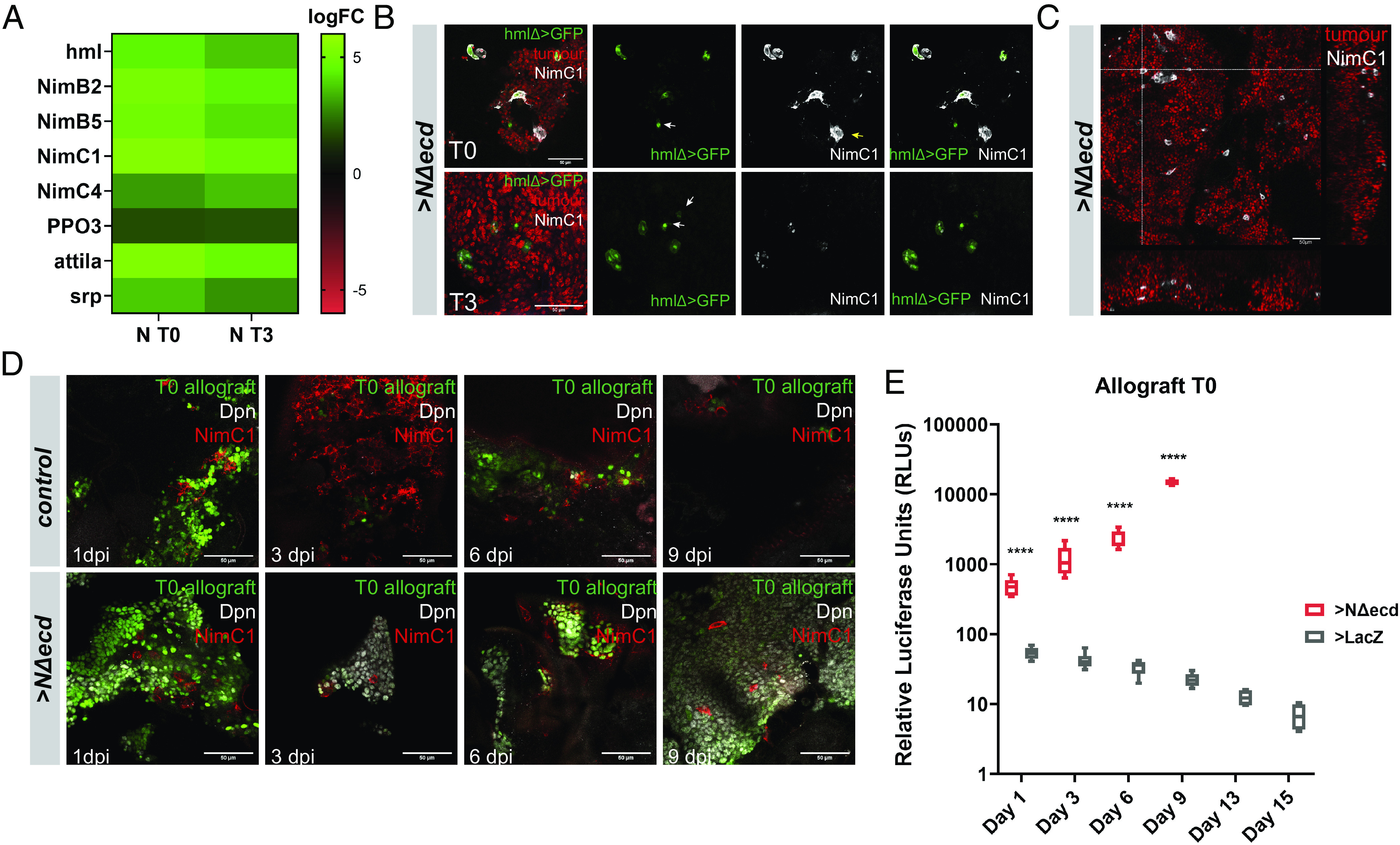
*NΔecd* allograft tumors are infiltrated by host hemocytes. (*A*) Selected hemocyte-related genes which were up-regulated in *NΔecd* allograft tumors (N-T0 or N-T3 vs. primary FACS). (*B*) Confocal images of T0 and T3 *grh^ts^>NΔecd* explants (red) transplanted into *hmlΔ>GFP* (green) hosts and stained for the phagocytic receptor NimC1 (white). White arrows: hmlΔ>GFP-positive only cells; yellow arrows: NimC1-positive only cells. (*C*) Confocal image of *grh^ts^>NΔecd* T0 tumor (red) with haemocytes stained for NimC1 (gray). Orthogonal sections of a confocal Z-stack (corresponding to the crosshairs) are shown. (*D*) Confocal images depicting hemocytes (NimC1, red) interacting with *actF/O* control or *NΔecd* brain allografts (marked with *UAS-GFP*, green) at the indicated dpi. Control fragments are gradually cleared off whereas *NΔecd* fragments continue to grow with hemocytes attached. Dpn (gray) marks NSC-like cells. (*E*) Box plot depicting relative luciferase activity levels from host flies carrying control (*grh^ts^ *>*lacZ*) or *grh^ts^>NΔecd* brain allografts (T0) over a time course of 15 dpi. No *NΔecd* samples were measured after 9 dpi as the tumor had killed hosts. Middle bars: median values; boxes: first to third IQRs; whiskers: 5% and 95%. ****P < 0.0001, (unpaired *t* test).

Processes related to immunity were notably enriched in *NΔecd* T0 and T3 transcriptomes compared to primary *NΔecd* CNS hyperplasias (Fig. 2 *B* and *E*). More specifically, genes normally expressed in hemocytes like the clotting factor *hemolectin* (*hml*) or phagocytic receptors of the Nimrod family were among a few hundred genes that were de novo turned on at the T0/T3 stage tumors (Fig. 4*A*). As allograft masses were not sorted before RNAseq, this could reflect tumor infiltration by host hemocytes, as previously reported for epithelial-derived allograft tumors (13). To test this hypothesis, we transplanted RFP-labeled *grh^ts^>NΔecd* tumors into hosts with GFP-labeled hemocytes [*hmlΔ-Gal4>UAS-GFP* (29)], grew them to T0 and T3 stages and stained for the phagocytic receptor NimC1. We indeed detected many hemocytes adhering and even penetrating into tumor masses extracted from host abdomens. (Fig. 4 *B* and *C*). The majority of tumor-associated hemocytes were double positive for hmlΔ>GFP and NimC1, with a few single positives (Fig. 4*B*, white and yellow arrows).

Unlike allografts, primary larval lesions did not seem to attract hemocytes (*SI Appendix*, Fig. S5 *A* and *B*). We hypothesized that superficial glia and their associated basement membrane (BM) enwrap the entire CNS and act as a blood–brain barrier that blocks hemocyte recruitment to the hyperplastic regions. We confirmed that *grh^ts^*>*NΔecd* tumors do not disrupt the larval CNS BM, which was visualized with a GFP-tagged collagen IV, vkg-GFP (*SI Appendix*, Fig. S5*C*). When we dissociated hyperplastic (or wt) larval brains and cocultured them with GFP-labeled hemocytes (*hmlΔ*>*GFP*) derived from larval bleeding, we observed hemocytes moving toward and attaching to NSC lineages (*SI Appendix*, Fig. S5 *D* and *E*). Thus, primary *NΔecd* hyperplasias do not attract hemocytes because they do not disrupt the larval CNS BM. Allografting severely damages the BM: A few days after transplantation it seems to be shed from the brain lobe and the exposed brain cells are associated with hemocytes (*SI Appendix*, Fig. S5*F*); this happens in both *wt* and *NΔecd* hyperplastic brains.

To explore the kinetics of hemocyte recruitment, we injected either control or *NΔecd* hyperplastic larval brain lobes into *w^1118^* hosts and performed a time-course of explant immunostainings with the hemocyte-specific antibody NimC1. We observed that both control and tumorous brain lobes triggered hemocyte recruitment as early as the first day post-injection (dpi) (Fig. 4*D*). By the third dpi, control cells were heavily surrounded and engulfed by hemocytes and by 6 dpi many of them were cleared. By day 9, only remnants of the original material or cellular debris could be detected (Fig. 4*D*). *NΔecd* brains, on the other hand, were not successfully cleared by hemocytes and instead they grew to amorphous and diffuse tumor masses that remained associated with hemocytes into 6 to 9 dpi. Eventually they killed their hosts by 10 to 12 dpi (Fig. 1*C*).

Finally, to quantitatively estimate the rate of growth or clearance of allografted tissue, we utilized a highly sensitive luciferase assay. We coexpressed a *UAS-luciferase* transgene (30, 31) along with *NΔecd* or *lacZ* in NSCs using *grh^ts^-Gal4* and subsequently transplanted these brain lobes into *w^1118^* hosts. Allografted whole-fly extracts were prepared over a period of 15 d, and luciferase activity was measured. Control allograft luciferase activity started declining on day 3 and continued to do so till the end of the experiment on day 15. On the contrary, luciferase activity increased exponentially in hosts transplanted with *NΔecd* brain lobes, until flies died by day 10 to 12 (Figs. 4*E* and 1*C*). Therefore, allografted foreign brain tissue can be efficiently cleared by the host, unless the graft contains tumorigenic cells, which grow at a fast pace and kill their hosts within a short period of time.

### Loss of Host hemocytes Accelerates Allograft Tumor Growth.

To test whether hemocytes assist or impede NSC-derived tumor growth, we performed hemocyte ablation on the hosts. To do this, we expressed the proapoptotic gene *hid* in the majority of larval hemocytes using the *hmlΔGal4; UAS-GFP* driver. Whole adult fly cryosections, tumor explant stainings, and hemocyte bleedings (*SI Appendix*, Fig. S6 *A*–*C*) revealed that *hmlΔ*>*hid+GFP* hosts had a reduced hemocyte population. The yield of hemocytes recovered by bleeding *hmlΔ*>*hid* adults was 1/3 of wt, with the surviving hemocytes being primarily hmlΔ-negative and NimC1-positive (*SI Appendix*, Fig. S6*D*).

**Fig. 5.**
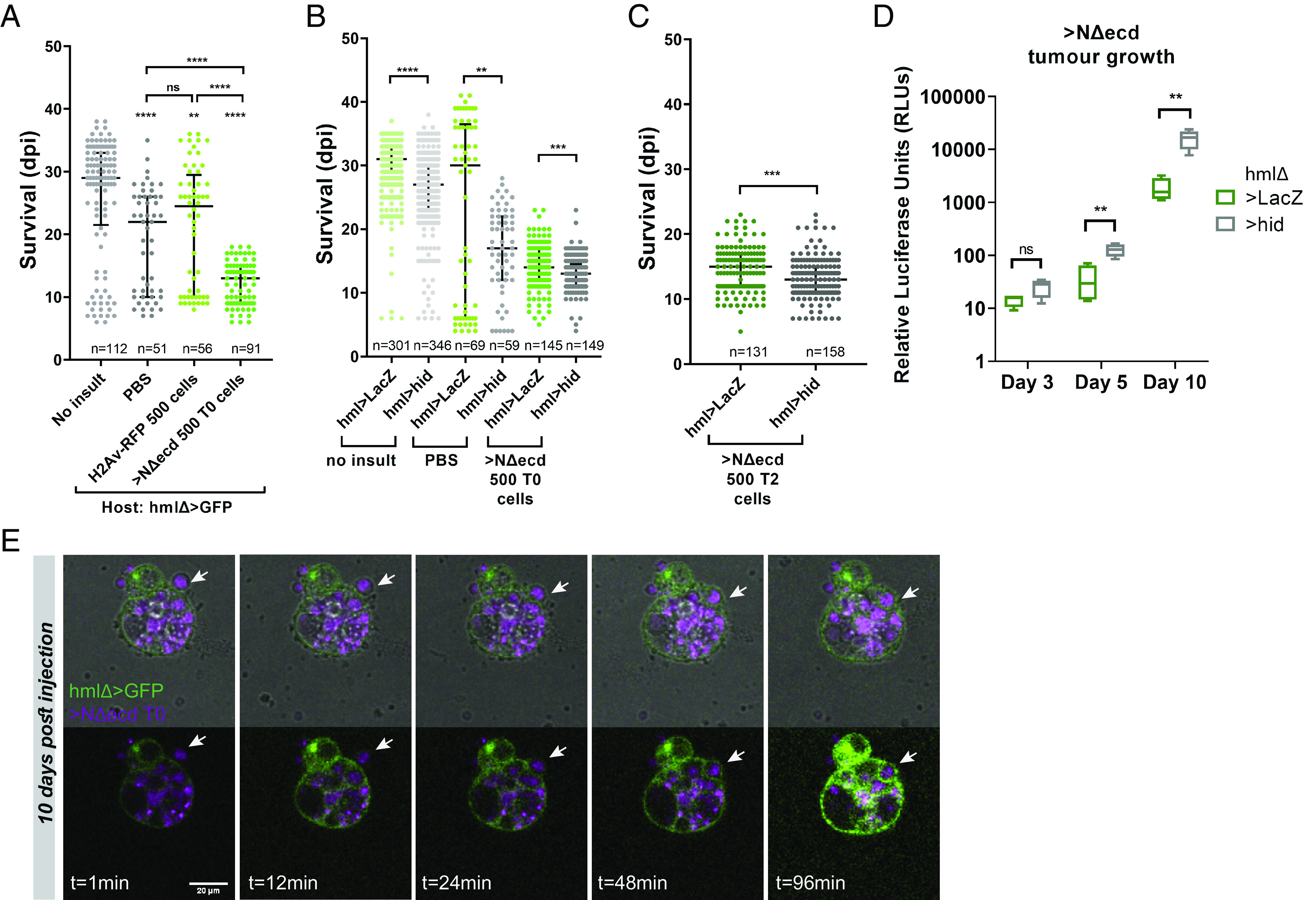
Hemocytes delay tumor growth and phagocytose tumor material. (*A*) Scatter plot indicating the lifespan of *hmlΔ*>GFP hosts either unchallenged (no insult) or injected with PBS, 500 control (H2Av-RFP) brain cells or 500 *grh^ts^>NΔecd* T0 cells. (*B* and *C*) Scatter plots with lifespan of the indicated host genotypes after no insult, PBS injection or transplantation of 500 *grh^ts^>NΔecd* T0 cells (*B*) or 500 *grh^ts^>NΔecd* T2 cells (*C*). Only RFP-positive hosts were scored; n = the total sample size pooled from 2 to 5 independent biological replicates. Middle black lines: median values; lower and upper black lines: first to third IQRs; **P_adj_ or *P* < 0.01; ***P_adj_ or *P* < 0.001; ****P_adj_ or *P* < 0.0001, ns: not significant (ordinary one-way ANOVA, Tukey’s multiple comparisons test for *A*; unpaired *t* test for *B* and *C*). (*D*) Box plot depicting luciferase activity from *hmlΔ>FP+LacZ* vs. *hmlΔ*>*GFP+hid* host flies carrying *grh^ts^>NΔecd+luc* allograft tumors (500 T0 cells) over a time course of 10 dpi. Middle bars: median values; boxes: first to third IQRs; whiskers: 5% and 95%. **P < 0.01; ns: not significant (unpaired *t* test). (*E*) Stills from a timelapse movie of *grh*^ts^*>NΔecd* tumor cells (magenta) raised in an *hml*>*GFP+lacZ* host (green hemocytes) for 10 dpi. (*Top*) fluorescent image superimposed on brightfield. White arrows follow a tumor cell being engulfed by the hemocyte. Note the big vacuoles/phagosomes inside the hemocyte carrying tumor debris from earlier phagocytic events. (Scale bar, 20 μm.)

First, we wanted to assess whether the genetic background of this new host (*hmlΔ*>*GFP*) affected its lifespan under various insults, namely injection of sterile PBS, wt brain cells or neoplastic *grh^ts^*>*NΔecd+RedStinger* cells (at 500 cells/host). Tumor cells caused a dramatic reduction in the host’s lifespan. A smaller effect on the host's lifespan was observed upon PBS or control cell injection, which were indistinguishable from each other (Fig. 5*A*).

We then generated hemocyte-depleted hosts (*hmlΔ*>*GFP, hid*) and injected them with PBS or *NΔecd* tumor cells (Fig. 5*B*). We detected a small but significant reduction in the survival of hemocyte-depleted hosts upon injection of either T0 (Fig. 5*B*) or T2 cells (Fig. 5*C*). However, a lifespan reduction was also observed upon PBS injection and even with no insult (Fig. 5*B*), implying that reduction of hemocytes compromises animal health, regardless of tumor.

We therefore turned to the *UAS-luciferase* assay to directly assess tumor burden. After injecting 500 T0 cells, we could detect a steady increase in luciferase activity from 3 to 10 dpi in both control and hemocyte-depleted hosts. At all-time points, hemocyte-depleted hosts displayed a higher tumor burden than controls (Fig. 5*D*). This points toward a tumor-suppressive role of adult hemocytes toward the *NΔecd* malignant cells.

### Host Hemocytes Associate with Tumor Cells and Phagocytose Them.

Since adult host hemocytes restrict tumor growth, we set out to explore how they achieve this. Hemocyte–tumor interactions were examined either in cryosections of allografted flies or in tumor explants. Tumor-bearing adult flies with GFP-labeled hemocytes were cryosectioned at specific time points post injection of 500 NΔecd-T0 cells. Interestingly, although tumor cells were injected at the posterior abdomen (*SI Appendix*, Fig. S6*E*, asterisks), they rapidly diffused through the hemolymph and were detected in the thorax even at 1 dpi (*SI Appendix*, Fig. S6 *E*-*I-1*). Tumor cells/clumps were frequently associated with hemocytes and tracheal structures (32) (*SI Appendix*, Fig. S6 *E* and *F*). We could detect several instances of hemocytes extending processes toward tumor cells (*SI Appendix*, Fig. S6 *E II-1* and *III-1’*) or engulfing them (*SI Appendix*, Fig. S6*E II-2* and *II-3* and *III-1’’*).

In order to confirm that hemocytes indeed phagocytose tumor cells, *NΔecd* allograft explants raised in hosts with GFP-labeled hemocytes were cultured and monitored in time lapse movies for several hours. Many highly vacuolated hemocytes were observed with fluorescent signal from tumor cells inside their phagosomes, indicating that these blood cells had already engulfed tumor material inside the host before explanting (Fig. 5*E*, *SI Appendix*, Fig. S6*G*, and Movie S1). During live imaging, hemocytes firmly attached to the dish surface, while tumor cells from the explant floated in the medium. When tumor cells collided with hemocytes, the latter captured them with their extended filopodia (*SI Appendix*, Fig. S6*G*, arrows). Once a tumor cell was captured by a hemocyte, it rarely escaped. Engulfment was observed on several occasions (Fig. 5*E*, arrows; Movie S1).

### Loss of Phagocytic Receptors and ROS (Reactive Oxygen Species) Signaling in Hemocytes Affects Tumor Growth.

**Fig. 6.**
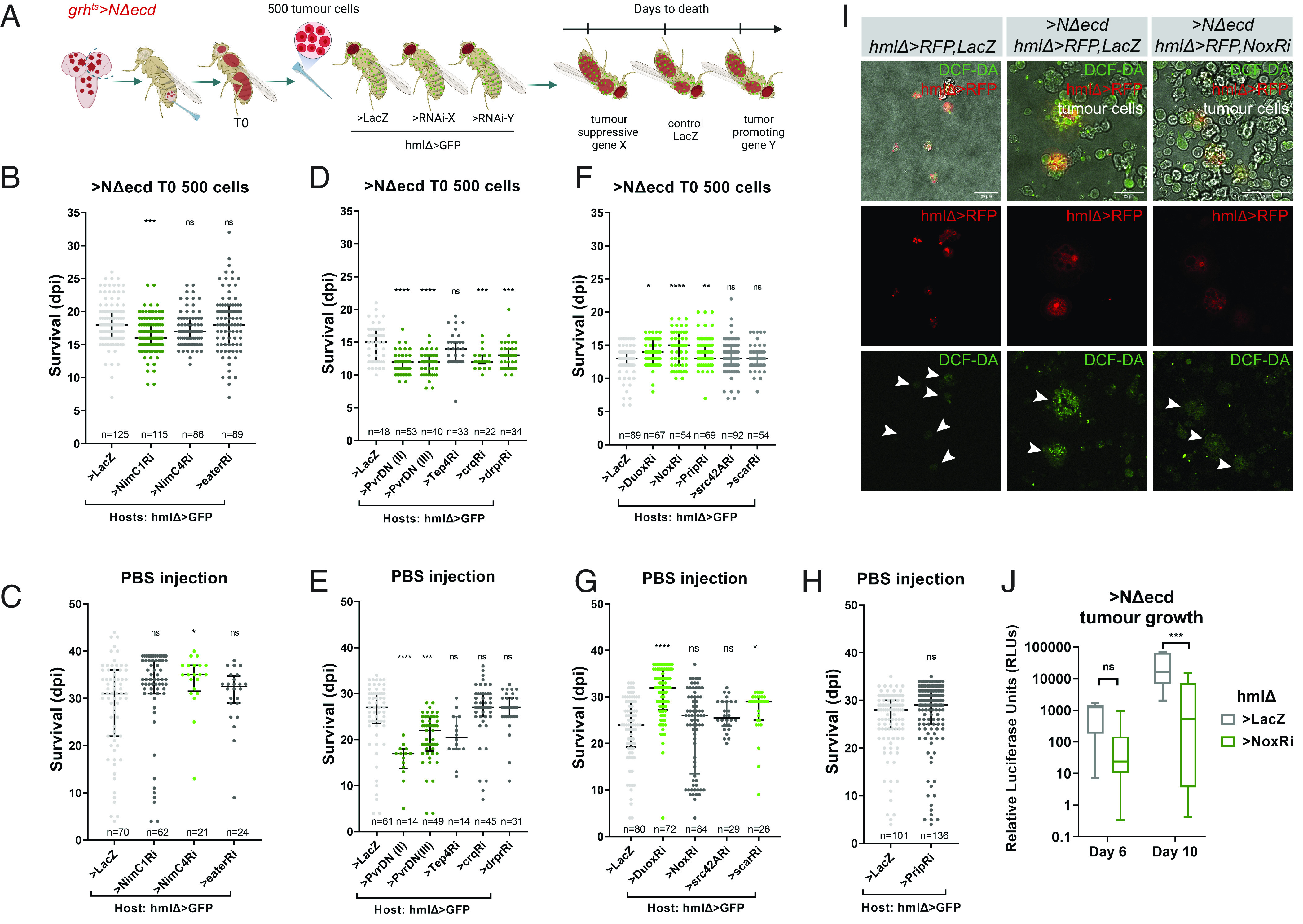
An RNAi screen in host hemocytes reveals that loss of phagocytic receptors accelerates tumor growth. (*A*) Cartoon of the RNAi screen in host hemocytes: *grh^ts^*>*RedStinger+NΔecd* brain lobes were transplanted into hemocyte-depleted hosts (T0). For T1: 500 T0 *> NΔecd* cells were retransplanted into hosts with genetically manipulated hemocytes or into controls (*hmlΔ*>*GFP +lacZ)*. (*B*–*Η*) Scatter plots showing the survival (in dpi) of hosts injected with 500 T0 *grh^ts^>NΔecd* cells (*B*, *D*, and *F*) or sterile PBS (*C*, *E*, *G*, and *H*). Only RFP-positive hosts were scored; n = the total sample size pooled from 2 to 5 independent biological replicates. Middle black lines: median values; lower and upper black lines: first to third IQRs; *P_adj_ or *P* < 0.05; **P_adj_ or *P* < 0.01, ***P_adj_ or *P* < 0.001; ****P_adj_ or *P* < 0.0001, ns: not significant (ordinary one-way ANOVA, Dunnett’s multiple comparisons test for *B*–*G*; unpaired *t* test for *H*). (*I*) Confocal images of either naïve control adult hemocytes (*hmlΔ*>*RFP,LacZ*) or tumor explants (*grh^ts^*>*NΔecd,* no fluorescent indicator) from normal (*hmlΔ*>*RFP,LacZ*) or *Nox* depleted (*hmlΔ*>*RFP,NoxRi*) hosts imaged live with the ROS indicator DCF-DA (green). Note that tumor-associated control hemocytes (arrowhead, *Middle*) have higher ROS levels than naïve hemocytes (arrowhead, *Left*) while *Nox RNAi* knockdown also reduces ROS levels in host hemocytes. Tumor cells also contain ROS at various levels. (Scale bar, 25 μm.) (*J*) Box plot depicting luciferase activity from *hmlΔ*>*GFP+lacZ* vs. *hmlΔ*>*GFP+Nox-RNAi* host flies carrying *grh^ts^*>*NΔecd+luc* allograft tumors (500 T0 cells) at 6 and 10 dpi. Middle bars: median values; boxes: first to third IQRs; whiskers: 5% and 95%. ****P < 0.0001 (unpaired *t* test).

To investigate the mechanisms that hemocytes exploit to resist *NΔecd* NSC tumor growth, we performed a hemocyte-targeted RNAi miniscreen. We injected 500 *NΔecd* T0 cells into hosts with either normal (*hmlΔGal4*>*GFP+lacZ*) or compromised hemocytes (*hmlΔGal4*>*GFP+ X-RNAi*, where X stands for one of 12 candidate genes). We then monitored for any differences in host survival as an indication of whether the hemocyte knockdown influences tumor-induced mortality (Fig. 6*A*). The viability of PBS injected and unchallenged hosts was also monitored as controls for the screen (Fig. 6 *C*, *E*, *G*, and *H* and *SI Appendix*, Fig. S7*A*). The 12 candidate genes screened are implicated in cellular immune responses like phagocytosis, hemocyte attraction to wounds, and ROS production.

First, we knocked down several genes encoding phagocytic receptors. These have been implicated in a multitude of processes, including defense against bacteria, engulfment of apoptotic corpses, sensing of chemoattractive damage signals (like ROS) and uptake of lipoproteins (33). Tumor-bearing hosts with hemocytes depleted of either *NimC1* (Fig. 6*B* and *SI Appendix*, Fig. S7*B*), *draper*, or *croquemort* (Fig. 6*D* and *SI Appendix*, Fig. S7*C*), had significantly poorer survival compared to control hosts. PBS-injected flies of the above genotypes were not significantly different from control (Fig. 6 *C* and *E*) showing that a control injection does not adversely affect survival of these flies. Loss of *eater* or *NimC4* in host hemocytes had no effect in their survival upon tumorigenic challenge (or PBS) (Fig. 6 *B* and *C*). We conclude that *NimC1, Croquemort* and *Draper* are used by hemocytes to impede tumor growth, probably by phagocytosis of tumor cells. This was confirmed by more detailed analysis of the *NimC1* knockdown hosts (see next section).

We next investigated whether perturbing the Pvr [Platelet-Derived Gowth Factor/ Vascular Endothelial Growth Factor (PDGF/VEGF) homologue] pathway in hemocytes would affect tumor growth. Pvf2, one of Pvr ligands, was up-regulated in *NΔecd* allograft tumors. Pvr, the receptor of the pathway, is localized on hemocyte cell membranes and responds to Pvf ligands during developmental migrations (34). Overexpression of a dominant-negative form of Pvr (*Pvr-DN*) in hemocytes resulted in a significantly reduced survival not only of tumor-injected hosts (Fig. 6*D*), but also of PBS-injected flies (Fig. 6*E*) or even unchallenged hosts (*SI Appendix*, Fig. S7*A*). We conclude that inhibiting hemocyte Pvr is generally detrimental to fly health but we cannot discern a role in tumor growth. We tested two more genes encoding the kinase Src42A and Scar both of which have been associated with hemocyte migration to wounds (35). Knockdown of *src42A* and *scar* had no significant effect on tumor-bearing flies (Fig. 6*F*) while upon PBS injection their silencing was slightly beneficial for the organism survival (Fig. 6*G*).

Another gene that was highly up-regulated in *NΔecd* allograft tumors was *tep4*. This gene encodes a secreted thioester containing protein known to act as an opsonin (a complement-like molecule that binds to microbes and facilitates their uptake by macrophages). Tep4 was found to be up-regulated in hemocytes upon bacterial infection (36–39). Knockdown of *tep4* in hemocytes did not result in changes in the survival of tumor-bearing or PBS-injected hosts (Fig. 6 *D* and *E*); however, Tep4-depleted flies died faster without any challenge (*SI Appendix*, Fig. S7*A*), suggesting that hemocyte Tep4 may affect the overall animal fitness.

Hemocytes respond to ROS and also produce ROS. Phagosome ROS play a role in pathogen killing (40), but ROS mediate a multitude of additional functions: hemocyte recruitment to sites of injury, where they participate in wound healing (41, 42) and recruitment to epithelial tumors where they may promote the overgrowth of cancer cells (11, 43). By using the fluorescent ROS-sensor DCF-DA, we found that *NΔecd* tumor-associated hemocytes had increased ROS levels compared to naïve adult hemocytes (Fig. 6*I*). We asked if hemocyte ROS sensing and production could also affect *NΔecd*-induced tumor growth. We tested the role of *Duox* and *Nox*, the two genes encoding NADPH oxidases that produce extracellular/endosomal ROS. *Nox* is up-regulated in N-T0/T3 transcriptomes, whereas *Duox* is down-regulated. We also tested *Prip*, a gene encoding an aquaporin-like channel that contributes to cytoplasmic accumulation of H_2_O_2_ (42). Interestingly, silencing of *Nox*, or *Prip* resulted in better host survival specifically upon tumorigenic challenge (Fig. 6*F*), and not after sterile PBS injection (Fig. 6 *G* and *H*). *Nox* RNAi resulted in decreased ROS levels in tumor-associated hemocytes (Fig. 6*I*). The luciferase assay validated that the tumor burden was significantly lower in *Nox*-depleted hosts compared to controls at 10 dpi (Fig. 6*J*). Therefore, hemocyte accumulation of ROS seems to enhance the mortality caused by the *NΔecd* NSC tumor. Silencing *Duox* in hemocytes also resulted in increased survival with tumor (Fig. 6*F*), but also after PBS injection (Fig. 6*G*), indicating that the beneficial *Duox* knockdown in hemocytes is not tumor specific. Therefore, we provide evidence of a tumor-promoting role of hemocyte-derived ROS production.

### Loss of *NimC1* in Hemocytes Affects Their Ability to Associate with Tumor Cells.

**Fig. 7.**
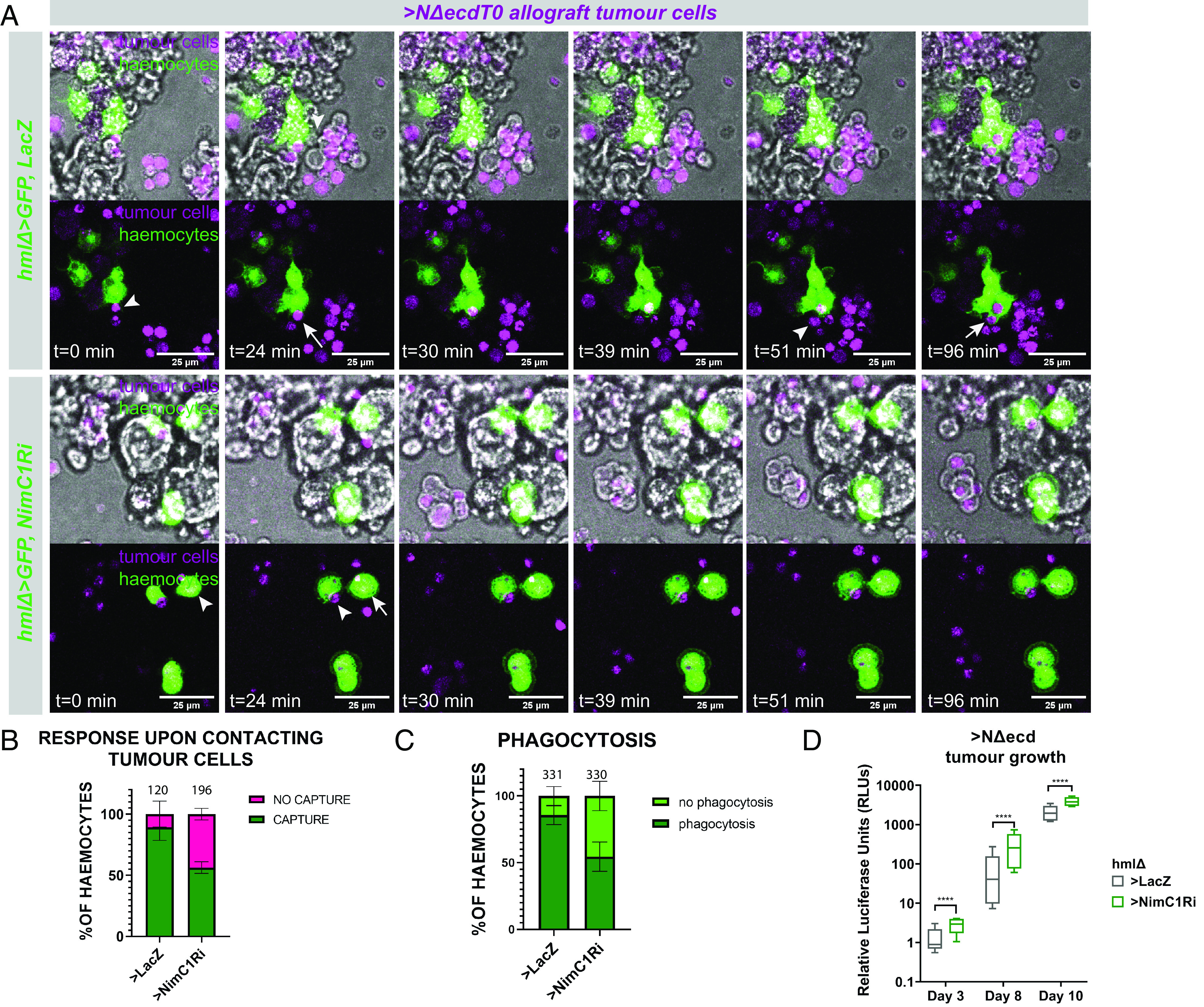
Hemocytes lacking *NimC1* cannot efficiently capture and phagocytose *NΔecd* tumor cells. (*A*) Stills from timelapse movies of *grh^ts^>NΔecd* allograft tumor cells (magenta) cocultured with naïve isolated hemocytes (green) from *hmlΔ*>*GFP+lacZ* (control) and *hmlΔ*>*GFP+NimC1-RNAi* adult flies. White arrows: tumor cells being engulfed by haemocytes, white arrowheads: tumor cells captured by hemocytes. (Scale bar, 25 μm.) (*B* and *C*) Diagrams depicting the proportion of hemocytes that capture tumor cells upon contact (*B*) or contain tumor material in their phagosomes after 4 to 6 h of coculture (*C*). The number of total contact events/hemocytes scored (from 3 biological replicates) is shown above each bar. Error bars indicate SD. (*D*) Box plot depicting luciferase activity from *hml*>*GFP+lacZ* vs. *hml*>*GFP+NimC1-RNAi* host flies carrying *grh^ts^>NΔecd+luc* allograft tumors (500 T0 cells) over a time course of 10 dpi. Middle bars: median values; boxes: first to third IQRs; whiskers: 5% and 95%. ****P < 0.0001 (unpaired *t* test).

Since the lifespan of hosts with *NimC1*-defective hemocytes was decreased upon challenge with NSC tumors, we set out to explore if this was due to inefficient phagocytosis of tumor cells. For this purpose, we performed ex vivo live imaging of cocultures of *NΔecd* explant allograft tumor or control larval CNS cells with bled adult Drosophila hemocytes that were either normal or depleted of *NimC1*. In these cocultures, hemocytes quickly adhered to the glass surface whereas tumor cells were floating in the culture medium randomly encountering hemocytes. These tumor–hemocyte encounters were recorded for 4 to 6 h with time lapse movies (Fig. 7*A*, *SI Appendix*, Fig. S7 *D* and *E*, and Movies S2 and S3). We observed that control hemocytes usually (in 90% of the encounters, Fig. 7*B*) extended filopodia, successfully capturing tumor cells and subsequently engulfed them (Fig. 7 *A*, *Top*, white arrows; Fig. 7*C* and Movie S2). On the other hand, *NimC1*-defective hemocytes exhibited various behaviors. Some appeared normal (circa 50%, Fig. 7*B*) as they extended filopodia, trapped and phagocytosed tumor cells (Fig. 7*A*, arrowhead; Fig. 7*C*, *SI Appendix*, Fig. S7*C*, and Movie S3). The remaining 50% (Fig. 7*B*) either did not respond to passing tumor cells or, when they did, they could not efficiently attach to (*SI Appendix*, Fig. S7*D* yellow arrowhead) or phagocytose (Fig. 7*A*; white arrowhead) the tumor cells, allowing the latter to escape back into the medium (Movie S3). *NimC1-*defective hemocytes’ response to control cells was similar to tumor cells but milder. Compared to normal hemocytes, a higher proportion of *NimC1*-defective haemocytes failed to capture (17% vs. 4%) or phagocytose (28% vs. 18%) control cells (*SI Appendix*, Fig. S7 *E*–*G*). Thus, we provide evidence that *NimC1*-defective hemocytes show an impaired ability to associate with control or tumor cells and phagocytose them.

To validate that the phagocytic defects seen in the live imaging experiments (Fig. 7 *A*–*C* and *SI Appendix*, Fig. S7*C*) and the decreased survival of *NimC1*-depleted hosts (Fig. 7*B*) correlate with increased tumor burden, we utilized the luciferase assay. At all timepoints, tumor-derived luciferase activity was significantly higher in *NimC1*-depleted hosts compared to controls (Fig. 7*D*). We therefore propose that phagocytosis defects caused by silencing of *NimC1* led to an increased tumor growth rate, which resulted in the accelerated death of the host.

## Discussion

Persistent Notch signaling or Hes expression in NSC progeny in Drosophila larvae leads to a block in differentiation, hyperplasia, and the generation of malignant tumors upon allografting. The transcriptome changes between host-propagated and primary larval tumors presented herein suggest that neurons and glia are eliminated after allografting. In addition, early differentiation-promoting factors, though not entirely repressed, continue to decrease from primary tumor to T0/T3. The transcriptome data are also indicative of a metabolic switch in both lipid and amino acid pathways and a multitude of stress responses. The growth regulator Myc is absolutely necessary for malignant conversion, since no allograft tumors could be recovered upon *Myc* knockdown (Fig. 3). The ability of overexpressed Myc to coordinately activate autophagy and antioxidant responses has been shown to underlie its overgrowth effects (44) and is consistent with our results, since both autophagy and antioxidants (gst genes) are up-regulated in Notch tumors. Depletion of the RNA-binding protein Imp also severely curtails *NΔecd* tumor growth. Besides stabilizing Myc RNA (28), Imp associates with many other mRNAs and has been shown to promote growth in another NB tumor, generated by the depletion of the prodifferentiation TF Pros (45, 46).

Two of the profound changes that happen upon allografting are the mechanical disruption of the CNS BM and the injury inflicted on the host’s epidermis by the transplantation procedure. Both of these events are known to stimulate hemocyte attraction. Indeed, we find that hemocytes attach and infiltrate the tumor (Fig. 4 and *SI Appendix*, Fig. S6*F*). These hemocytes often contain tumor material in their phagosomes. Tumor phagocytosis appears to slow down the tumor’s growth, as evidenced by either depleting the hemocyte population or disabling their phagocytic ability by knocking down photoreceptors, like NimC1. Both of these manipulations accelerate tumor growth and hasten the demise of the host. We do not think that the hemocytes’ response is specific to the *NΔecd*-tumor, as we have documented a similar response to nonmalignant brain lobes after allografting (Fig. 4 and *SI Appendix*, Fig. S5*F*) or upon coculture (*SI Appendix*, Figs. S5*D* and S7*E*). It is possible that hemocytes attack any foreign tissue fragment injected at the wound, but the tumor simply evades destruction by these immune cells by virtue of its ability to grow rapidly.

Over the past two decades, many eye or wing disc-derived tumor studies have given us insight on the changes that underlie malignant transformation. A common denominator in these epithelial cancers is activation of JNK, a stress-responsive proapoptotic pathway, coupled with activation of oncogenes, which, among others, leads to inhibition of apoptosis (43, 47–51). The *NΔecd*-tumor transcriptome contains most core components of the JNK pathway, but shows no evidence of increased JNK activity e.g., upregulation of *puc* or *Mmp1* (48) in the allografts vs. primary tumors. Also, expression of the TNF cytokine *egr*, a major upstream activator of the JNK cascade, is reduced (but still detectable) after allografting. Other JNK targets are the Upd cytokines that activate the Jak/STAT pathway and may act as growth factors within epithelial tumors (13). However, none of the three *upd* genes are detected in the *NΔecd*-tumor transcriptomes. Therefore, neither JNK signaling nor Jak/STAT are at a high level within *NΔecd* tumors; instead these neural malignancies seem to use different oncogenic strategies than epithelial tumors to overgrow and colonize their hosts. Epithelial tumors also induce cachexia to their hosts, hallmarked by organ wasting and fluid retention (8); we never observed such systemic effects in the *NΔecd* NB tumor bearing hosts.

A common feature of tumors and wounds is the accumulation of ROS generated by damaged cells or actively produced extracellularly by wound-associated hemocytes that express the membrane-associated oxidases Nox and/or Duox. Some extracellular ROS species, primarily H_2_O_2_, are known to recruit blood cells to wounds in both mammals and flies; these cells help clear cellular debris and repair the injury. The *NΔecd* tumor-associated hemocytes contain high ROS, and this somehow increases the morbidity of the fly—either by helping the tumor grow faster or by generating a widespread inflammation which hastens the host’s death by the tumor.

Human NSC tumors, share many characteristics with the herein described N-tumor. Myc plays an important role in the initiation and progression of certain types of medulloblastomas and gliomas (52, 53). The similarities extend to the reliance of human tumors on insulin/Insulin-like Growth Factor (IGF) signaling. IGF1R activation has been implicated in increasing tumor aggressiveness. Notch has also been implicated in glioma growth (54). In human glioblastoma explanted cultures mutant for *TRIM3*, transport of active NOTCH1 (NICD) is perturbed, resulting in enhanced tumor growth (55). A population of Notch-dependent slow-growing cancer stem cells has been implicated in the drug resistance [to Receptor Tyrosine Kinase (RTK) inhibitors] of certain types of glioblastoma (56). Rare subclones acquire further genetic changes which activate insulin and AKT signaling programs enabling their rapid expansion over time (57). Thus, the interplay of insulin/IGF, Myc, Notch and HES seems to be a common intrinsic axis controlling NSC carcinogenesis.

Finally, Notch regulates coevolution of malignant glioma and immune cells in the TME. In early stages of GBM, glioma cells increase their aggressiveness and avoid immune surveillance by reducing Notch signaling, which results in recruitment of immunosuppressive TAMs, instead of antitumor TAMs and T cells (58).

Similar tumor-intrinsic mechanisms and interactions with the microenvironment seem to shape brain tumor progression in flies and mammals. The easily manipulable fly system that we have described may help devise intervention strategies to treat these highly lethal tumors.

## Materials and Methods

### Drosophila Strains and Genetics.

Drosophila stocks and crosses were maintained in standard conditions (details in *SI Appendix*).

Hyperplastic or control larval CNSs were obtained either with the act-F/O or the *grh^ts^*system. See *SI Appendix*, *Materials and Methods* for details.

Host flies’ preparation for the hemocyte ablation and RNAi screen experiments as well as for bleedings and live imaging experiments are described in *SI Appendix*.

### Transplantation Procedure.

Transplantations were performed as previously described by ref. 20 and as recently summarized in refs. 24, 25, and 59; for detailed description see *SI Appendix*, *Materials and Methods*.

Hosts were mesoscopically examined daily for viability (survival assay) and GFP/RFP signal detection under an epifluorescent stereoscope.

### Immunohistochemistry.

Fixation and immunohistochemistry of larval tissues were performed according to standard protocols (60), see *SI Appendix*, *Materials and Methods and Materials Table*.

### Live Imaging.

Both cocultures of larval brains with larval hemocytes and tumor explant with adult hemocytes (freshly isolated by perfusion as described in ref. 61 were prepared as described in *SI Appendix, Materials and Methods*.

All imaging was performed on a Leica TCS SP8 microscope (FORTH-IMBB confocal facility).

### Luciferase Assay.

Luciferase assays in tumor-bearing flies were performed as previously described (30, 31) using a Luciferase Assay Kit [Promega (details in *SI Appendix*, *Materials and Methods*)].

### FACS Purification, RNA prep, RNA-seq Library.

NSC-like cells from circa 150 hand-dissected larval CNSs were dissociated and isolated by FACS according to published protocols (62) and as previously described in ref. 25. Tumor cells from T0 or T3 stage (25 hosts, circa 10 dpi) were collected in ice cold PBS.

RNA was extracted with Trizol according to standard protocols from FACS sorted cancer NSCs or T0 and T3 allograft tumors (three replicates per sample) and was subsequently used for the RNA-seq library preparation.

NGS libraries were generated using the polyA mRNA magnetic isolation kit [New England Biolabs (NEB)] and the NEB Ultra II RNA library kit for Illumina kit according to manufacturer’s protocol, using 13 cycles of amplification. Libraries were sequenced on Illumina Nextseq 500 on 1 × 75 High flowcell. (More details in *SI Appendix*, *Materials and Methods*).

## Supplementary Material

Appendix 01 (PDF)

Dataset S01 (XLSX)

Dataset S02 (XLSX)

Dataset S03 (XLSX)

Dataset S04 (XLSX)

Movie S1. *grh^ts^>NΔecd* tumour cells (magenta) raised in an *hml>GFP+lacZ* host (green haemocytes) for 10 days prior to explanting and imaging live. Timelapses were captured every 2min for a duration of circa 4 hours. Top: fluorescent image superimposed on brightfield to visualize cell morphology. Scale bar 20μm. Movie is related to Fig.5E.

Movie S2. *grh^ts^>NΔecd* allograft tumour cells (magenta) co-cultured with naïve isolated adult haemocytes (green) from *hmlΔ>GFP+lacZ* (control). Timelapses were captured every 3min for a duration of circa 4-6 hours. Top: fluorescent image superimposed on brightfield to visualize cell morphology. Note the highly motile filopodia emanating from the haemocytes. Scale bar 25μm. Movie is related to Fig.7A.

Movie S3. *grh^ts^>NΔecd* allograft tumour cells (magenta) co-cultured with naïve isolated adult haemocytes (green) from *hmlΔ>GFP+NimC1-RNAi*. Timelapses were captured every 3min for a duration of circa 4-6 hours. Top: fluorescent image superimposed on brightfield to visualize cell morphology. Scale bar 25μm. Movie is related to Fig.7B.

## Data Availability

RNA seq raw data have been deposited in GEO (GSE219067) (63). Previously published data were used for comparisons with the datasets produced in this work (https://doi.org/10.1242/dev.126326, https://doi.org/10.1387/ijdb.210187cd, https://doi.org/10.1242/dev.191544, https://doi.org/10.1126/science.1195481, doi: 10.1016/j.celrep.2012.07.008) (27, 64–67). Requests for resources and reagents should be directed to Christos Delidakis (delidaki@imbb.forth.gr) or Eva Zacharioudaki (Evanthia_zacharioudaki@imbb.forth.gr).
