# Supplementary material for: Growth deregulation and interaction with host hemocytes contribute to tumor progression in a Drosophila brain tumor model
Source: Proc Natl Acad Sci U S A. 2023 Aug 7;120(33):e2221601120. doi: 10.1073/pnas.2221601120 (PMC10438840; doi:10.1073/pnas.2221601120)
Supplement: Supplementary file 1 — Appendix 01 (PDF) [file pnas.2221601120.sapp.pdf]

PNAS

Supporting Information for:

Growth deregulation and interaction with host haemocytes contribute to tumour progression in a Drosophila brain tumour model

Chrysanthi Voutyraki[1,2], Alexandros Choromidis[1,2], Anastasia Meligkounaki[1,2], Nikolaos Andreas Vlachopoulos[1,2], Vasiliki Theodorou[1], Sofia Grammenoudi[3], Emmanouil Athanasiadis[4,5], Sara Monticelli[6], Angela Giangrande[6], Christos Delidakis[1,2, *], Evanthia Zacharioudaki[1, *]

* Co-corresponding authors: Christos Delidakis and Evanthia Zacharioudaki.

Email: delidaki@imbb.forth.gr & evanthia_zacharioudaki@imbb.forth.gr:

This PDF file includes:

Supporting Materials and Methods
Supplementary Figures S1 to S7
Materials Table
Legends for Datasets S1 to S4
Legends for Movies S1 to S3
SI References

Other supporting materials for this manuscript include the following:

Datasets S1 to S4
Movies S1 to S3

Supporting Information Appendix

Materials and Methods

Drosophila strains and genetics

Drosophila stocks and crosses were maintained in standard cornmeal/yeast medium at 25°C or 18°C with a 12/12 light/dark cycle. The strains used are described in the Materials Table below.

Hyperplastic larval CNSs were obtained either by using the flip-out system or a temporally controlled progenitor-specific driver. In the first case, male flies from the *hs-FLP; act-FRT>STOP>FRT-Gal4, UAS-nlsGFP* stock (*act-F/O* in brief) were crossed with females of *UAS-NΔecd* [1] or *UAS-LacZ* (control). Their progeny underwent heat shock for 1 hour at 37°C at 72 h after egg laying (AEL). Upon heat shock, the Flippase (FLP) was produced and randomly catalyzed the excision of an FRT flanked polyadenylation site (transcriptional STOP) upstream of *Gal4*. As a result, Gal4 was produced from an actin promoter in random cells and their progeny (clonal constitutive expression) and drove the expression of all UAS transgenes (*GFP* and *NΔecd* or *LacZ*. Thus, GFP marked *act-Gal4* (flip-out) clones were generated. CNSs were dissected out from wandering third instar larvae 4 days after clone induction and used for transplantation. For the temporally regulated, progenitor-specific expression of *NΔecd*, flies carrying *UAS-NΔecd* or *UAS-LacZ* (control) were crossed with *tubP-Gal80^{ts}, UAS-RedStinger/CyO, Tb;grhNB-Gal4/TM6B* (referred to as *grh^{ts}-Gal4*) stock [2]. α*Tub-Ga80ts* was used to temporally control the expression of transgenes. Progeny were maintained at 18°C for 10 days where Gal80^{ts} was inhibiting Gal4, then shifted to 29°C for 48 h, where Gal80^{ts} was inactivated and *grhNB-Gal4* was

permitted to drive the expression of *UAS* transgenes. Subsequently, larval CNS were dissected out and used for further experiments.

For the functional experiments with *Myc*, *InR* (constitutively active and dominant negative forms), and *Imp*, the respective transgenes were combined with *UAS-NΔecd* or *UAS-LacZ* (control) lines and *act-F/O* tumours were generated as described above. A neutral transgene, usually *UAS-w-RNAi*, was used as a negative control to balance the dosage of total *UAS* transgenes per genotype.

For quantitatively scoring hyperplasias in the larval CNS, the experimental regimens were slightly altered to create milder hyperplasias (thus allowing easier manual counting of the hyperplastic vs normal clones); for the *act-F/O* system, larvae of the appropriate genotypes underwent heat-shock for 35min at 37°C (72 hours AEL) and CNSs were dissected out 3 days later.

For collecting larval haemocytes, *hmlΔGal4, UAS-2xEGFP* larvae were used. For collecting adult haemocytes, *hmlΔGal4, UAS-2xEGFP* flies were crossed to appropriate responder UAS lines and their F1 progeny were bled as detailed in the "Live Imaging" section.

Host flies for the haemocyte ablation and the RNAi screen experiments were prepared as follows: female flies from *hmlΔGal4, UAS-2xEGFP* (BL#30140) were crossed with *UAS-hid/CyO,Tb*, or *UAS-lacZ* or the respective RNAi lines obtained from Bloomington (Materials table). Crosses were maintained at 18°C to keep Gal4 activity low during larval/ early pupal stages (hmlΔGal4 is not expressed at the embryo stage, anyway [3]). At late pupal stage (16-18 days after egg laying), progeny was shifted to 29°C to boost Gal4 activity. Adult flies circa 5-6 days after adult eclosion were injected with PBS or transplanted with 500 control brain cells or 500 grh^{ts}>*UAS-NΔecd* T0 or T2 cells (haemocytes ablation experiments). Adult flies either challenged as described above or unchallenged were monitored until their death.

Transplantation procedure and survival assay

Transplantations were performed as previously described by [4] and as recently summarized in [5]–[7]. To generate brain tumours, we utilized either the *act-F/O* or grh^{ts} system to either drive *UAS-NΔecd*, *UAS-LacZ* (control) or combinations of transgenes (*UAS-NΔecd* + *UAS-RNAi* lines for genes of interest) as described above. Larval CNSs were dissected and sliced into individual brain lobes. Each brain lobe was loaded into a fine glass needle and transplanted into the abdomen of adult female fly hosts (w^{1118}, unless otherwise stated) using a Nanoject II Auto-Nanoliter Injector (Drummond Scientific Company; Cat# 3-000-205A). All female fly hosts were of similar age at the day of the transplantation i.e 3-4 days after adult eclosion except for the host flies used for the haemocyte ablation and the RNAi screen experiments which were all 5-6 days after adult eclosion to allow haemocytes to complete the clearance of larval fat body cells and relocate to adult tissues [8]. After the transplantation, flies were placed in fresh vials and were kept at 25°C (*act-F/O* system) or 29°C (grh^{ts} system) in a horizontal position. Fly food was renewed every 2-3 days to keep injected flies clean and reduce the load of potentially harmful germs growing in the vials. Hosts were macroscopically examined on a daily basis for viability (survival assay) and GFP/RFP signal detection in the abdomen or other parts of their body (thorax, head) under an epifluorescent stereoscope. Animals that died within three days post injection were excluded from the survival analysis as mortality could be attributed to damage during the injection procedure and not by the graft. Occasionally, some host animals continued to die up to days 7 post injection. When macroscopically examined post mortem, they had developed some tumour burden which may or may not have been the cause of their death. For the survival assays, we only scored hosts that developed detectable tumour (GFP/RFP), unless otherwise stated. For control animals bearing no insult we did not exclude any deaths from the analysis. Even in these control cases, occasional

animals died during the 10 first days post eclosion in some replicates for reasons that we do not understand. The day of death of each fly host was depicted as an individual dot in Survival Scatter plot diagrams (see below the “Quantification and Statistical analysis” section).

5-15 days after transplantation, tumour (derived from the transplantation of the hyperplastic brain tissue; T0) was dissected out of the fly host into a glass slide filled with 1X sterile PBS by performing an epidermal incision in the abdominal area. The material was either used for re-transplantation (serially for up to three more passages–T3), immunohistochemistry and live imaging experiments or was stored in Trizol for RNA extraction and library preparation for RNA seq analysis. To ensure that a consistent number of tumour cells was injected in the allograft passages (T1-T3), tumour pieces from the previous stage (T_{n-1}) were dissected out, dissociated in sterile PBS to single cell suspension (mechanically, via repeated aspiration through an insulin needle), counted on a haemocytometer and resuspended in filtered PBS at the desirable cell density. For the RNAi screen, we injected 500 tumour cells or sterile PBS at 36.8 nl per fly.

Immunohistochemistry

Fixation and immunohistochemistry of larval tissues were performed according to standard protocols [9]. Allograft tumours were fixed and stained as previously described [5]. After separating the abdomen from the rest of the fly body into a glass plate filled with 1X PBS, a slight cut was performed ventrally and fixation into 4% formaldehyde followed for 35 min at RT. After blocking in PBT (1X PBS, 5% BSA, 0.1% Triton) for at least 2h or O/N, tissues were incubated in primary antibody O/N at 4°C in a 96-well plate. After three 10-minute washes with 1X PT (1X PBS, Triton 0.1%), an O/N incubation with secondary antibodies and Hoechst followed. After breaking up the abdomen to fragments of internal organs mixed with tumour, we mounted the

samples in glass microscopy slides in mounting medium (80% glycerol with 0.5% N-propyl gallate).

Primary and secondary antibodies used are described in the Materials table. Samples were imaged using a Leica SP8 confocal microscope at the FORTH-IMBB confocal imaging facility.

Live imaging

For larval brain co-cultures with larval haemocytes: Larval brains were dissected in Complete Schneider's Culture Medium [Schneider medium (GIBCO; Cat# 21720024) supplemented with 1 mg/ml glucose (D-L- glucose monohydrate), 10% FBS (F4135), 4% larval extract, 1 mg/ml human insulin (Sigma; Cat# I9278) and 1x Antibiotic Antimycotic (GIBCO; Cat# 15240062)]. CNSs were dissociated in collagenase solution [2 mg/ml (Sigma; Cat# C0130)] for 20 minutes at RT, rinsed with culture medium and mechanically sheared using a 200μl pipette tip (pipetting up and down). Clumps of dissociated CNS cells were transferred on a glass-bottom Mattek dish (Cat# P35G-1.5-14-C), where they were left to settle for 15 minutes. Larval haemocytes were collected in ice cold culture medium by making an incision in the ventral part of the larval body and gently rubbing the cuticle to detach most of the tissue-resident haemocytes. A total of ten larvae were used per experiment. Haemocytes were added on top of the CNS culture prior to imaging. Samples were immediately imaged for 4-5 hours on a Leica SP8 confocal microscope at RT ~22°C (FORTH-IMBB confocal facility).

For tumour explant cultures and co-cultures with adult haemocytes: Tumour explants were dissected out on a glass slide in 300μl culture medium and were gently dissociated to clumps by pipetting up and down using a yellow pipette tip. Explants were either imaged immediately after isolation (to observe the tumour-resident haemocytes) or were co-cultured with freshly isolated

adult haemocytes. Adult haemocytes were collected with perfusion as previously described [10]. An insulin syringe was loaded with culture medium and a fine glass capillary needle (same needle used for the nanoinjector) was attached to the needle. A small incision was made at the posterior ventral side of the abdomen of a cold anaesthetized female and the tip of the glass capillary was inserted into the lateral thorax. Culture medium was gently perfused in the fly and 5 drops/fly coming out from the abdominal cut were collected on a Mattek dish. A total of ten adult flies were bled for each experiment. Tumour suspension was subsequently (10-15 min later) added on top of the haemocyte population prior to imaging. Samples were immediately imaged for 4-6 hours on a Leica TCS SP8 confocal microscope (FORTH-IMBB).

2′,7′-Dichlorofluorescin diacetate (DCF-DA) staining: Tumour explant was isolated in unsupplemented Schneider's medium and DCF-DA (Sigma; Cat# D6883) was added at final concentration of 1mg/ml. Samples were imaged live 30 minutes later on a Leica SP8 confocal microscope (FORTH-IMBB confocal facility).

Luciferase assay

Luciferase assays in tumour-bearing flies were performed as previously described [11], [12] Luciferase activity was measured using a Luciferase Assay Kit (Promega; Cat# E1500). First, we generated flies carrying both *UAS-NΔecd* (or control *UAS-lacZ*) and *UAS-luciferase* transgenes. Tumourigenic (*UAS-NΔecd/tubP-Gal80^{ts}, UAS-RedStinger; UAS-luciferase/grhNB-Gal4)* or control (*UAS-lacZ/tubP-Gal80^{ts}, UAS-RedStinger; UAS-luciferase/ grhNB-Gal4*) brain lobes were transplanted into *w^{1118}* hosts. For some luciferase experiments, tumours were serially re-transplanted to the T1 stage (500 cells injected). Three flies were used per biological replicate and a total of 3-4 biological replicates was collected (9-12 flies) for each genotype and timepoint.

Whole fly extract was prepared in 1X Passive Lysis Buffer using Kontes pestles and Eppendorf tubes. The extracts were centrifuged twice at 10.000g for 15 minutes at 4°C and the supernatant was stored at -20°C. Luciferase activity was measured using a Luminometer (Turner Designs; Cat# TD-20210) by adding Luciferin substrate (LARII; provided in the kit) to each sample. Normalization of Luciferase levels was performed by measuring total protein content in the samples by Bradford assay.

Cryosections

Adult tumour-bearing flies were anesthetized on ice and quickly washed in EtOH on a glass slide. To create an opening for the fixative, flies were placed on 1X PBS and the proboscis was removed. Fixation with 4% formaldehyde followed for 2.5h at RT and after two washes with 1X PBS, flies were incubated in 25% sucrose solution O/N at 4°C. Flies were blotted dry on tissue paper and embedded into OCT (Tissue-Tek Cat# 4583) containing cryocubes, frozen instantly on dry-ice/ethanol, and kept at -80°C until sectioning. Sections were obtained at -25°C on a Leica CM1850 cryostat with a section thickness of 30-50μm. Frozen sections were transferred on Superfrost Plus Microscope Slides (Menzel; Cat# J1800AMNZ) and stored at -20°C overnight. Next day, sections were postfixed for 12 minutes with 4% formaldehyde at RT. After 3 gentle rinses with 1X PBS, samples were mounted with Drop-n-Stain EverBrite™ Mounting Medium (BIOTIUM, Cat# 23009), covered with 24X50mm coverslips and sealed with nail polish. For every genotype/timepoint at least two flies were used.

FACS purification, RNA prep, RNA-seq library

Neural stem cell-like cells from larval brains were dissociated and isolated by FACS according to published protocols [13] and as previously described in [5]. Hyperplastic CNSs from 150 larvae

bearing GFP positive *act-F/O* clones overexpressing *UAS- NΔecd* were dissected out in ice cold 1x Rinaldini solution (Rinaldini 10X: 1.4M NaCl, 26 mM KCl, 4 mM NaH2PO4, 120 mM NAHCO3 and 50 mM Glucose) and incubated in Dissociation Solution [Collagenase 2 mg/ml (Sigma, Cat# C0130) in Complete Schneider's Culture Medium]. After three washes in 1x Rinaldini to remove Collagenase remnants, larval brains were gently disrupted in 200µl of 1x Rinaldini supplemented with 10% FBS. The cell suspension was filtered through a cell strainer (30µm mesh) into a 5ml FACS tube (Corning; Cat# 352235) filled with 800µl of 1x Rinaldini and 10% FBS. Propidium Iodide was added to the sample at a final concentration of 0.5µg/ml prior to sorting to exclude dead cells. NSC-like cells were isolated on a BD FACS ARIA III using a 100-micron diameter nozzle at a sheath pressure of 20psi. Live, GFP-positive cells of which the majority were cancer NSCs (there were also a few mature neurons and glia) were sorted according to the gating strategy described in [5]. Cells were sorted at an event rate of a maximum of 6,000 events per second into 15ml falcon tubes containing 1ml of 1x Rinaldini solution. Isolated cells were centrifuged at 300g for 5 min and the pellet was resuspended in 500µl of Trizol and instantly frozen. The sample was named N-FACS.

Tumour cells at the T0 or T3 stage which are very diffuse and break up easily to single cells/ small clumps were collected in ice cold PBS from hosts circa 10 days post injection and stored frozen in Trizol. These samples were named N-T0 and N-T3.

RNA was extracted with Trizol according to standard protocols from FACS sorted cancer NSCs of 150 animals (approx. 1,000,000 cells) or allograft tumours at T0 and T3 stages grown in 25 adult host flies (approx. 2,500,000 cells) per replicate. Three replicates per sample (N-FACS, N-T0 and N-T3), nine RNA preps in total, were subsequently used for the RNA-seq library preparation.

NGS libraries were generated using total RNA as input with polyA mRNA magnetic isolation kit (NEB) and the NEB Ultra II RNA library kit for Illumina kit according to manufacturer's protocol, using 13 cycles of amplification. Libraries were sequenced on Illumina Nextseq 500 on 1 x 75 High flowcell.

Quantification and statistical analysis

Quality control checks on raw *fastq* sequence data was performed by *FastQC*[14] (Version 0.11.9). Generated *fastq* files were aligned to the *Drosophila melanogaster* reference genome (Genome assembly: BDGP6.32) using *STAR* [15](Version 2.7.5), while raw counts were created using the *featureCounts* [16](Version 2.0.1). The original data can be retrieved from NCBI GEO under accession Number GSE219067. Downstream analysis of the resulted counts was performed in *R* [17](Version 4.0.2) using the standard *DESeq2* [18] (Version 1.30.1) package pipeline. The DESeq2 analysis is presented in Figures 2 and S2. DEG genes from pairwise RNAseq comparisons were filtered for $|log_2FC| \geq 0.5$, $p_{adj} \leq 0.05$, base mean $\geq$30. These lists were used as input in Metascape [19] to search for significantly enriched terms and the results are shown in Fig. 2 and S2.

For the correlation heatmap with a dendrogram (Fig S2. A), N and DM (FACs or T0, T3) sample distances were calculated using the dist function (DEseq2 package) to the transpose of the variance stabilizing transformed (VST) count matrix, while, heatmaps were created using the pheatmap R package (Version 1.0.12) with the default parameters.

For Figure S3, differentially expressed genes with $|log_2FC| \geq 0.5$, $p_{adj} \leq 0.05$ were used as an input to generate heatmaps to search for similarities with microarray transcriptome data from other allograft brain derived tumours originating from genetic insults in the asymmetric cell division

machinery [20]. They were also used as an input to generate venn diagrams to look for similarities with genes enriched in normal NSCs and neurons [21].

For Figure S1.B, the deep learning network StarDist was used to identify Pros+ve and Dpn+ve nuclei in single image channels and these nuclei were subsequently tracked in 3D in allograft T0, T1, T2 and T3 *NΔecd* tumour pieces using the Trackmate plugin in Fiji [22], [23]. Tracks were manually corrected to identify single nuclei throughout different z stacks. Each tracked tumour allograft piece contained 1500-3500 nuclei. The percent of Pros+ve cells over total tumour cells (defined as the number of Pros+ve nuclei divided by the sum of Dpn+ve and Pros+ve cells per 3D image) were subsequently plotted.

The statistical analysis of different genotypes was performed in Graphpad Prism 8 by using i) either student *t*-test, for comparing two genotypes/conditions ii) ordinary one-way ANOVA, adjusting P values with either Dunnett's multiple comparisons test when comparing multiple genotypes with one control or with Tukey's multiple comparisons test when comparing all genotypes/conditions between them. Scatter plot diagrams in all figures depict all data points (with each data point representing one host fly) pooled from 2-5 independent biological replicates with median values (middle black lines) and first to third interquartile ranges (IQRs: lower and upper black lines, 25th and 75th percentile, respectively); *p or adjusted p values are indicated as following: *P<0.05; **P<0.01; ***P<0.001; ****P<0.0001, ns stands for not significant.* Box-plot diagrams in all figures depict the median values (middle bars) and first to third interquartile ranges (boxes); whiskers are 5% and 95%; dots indicate outliers. p values are indicated as following: **P<0.05; **P<0.01; ***P<0.001; ****P<0.0001, ns stands for not significant.* Error bars in Figure 7 represent the standard deviation. All details of statistical analyses, including

numbers (n=) of the sample size scored for each genotype/condition, are found in the figure panels or figure legends.

All images from the genetic experiments were analysed using Fiji and Photoshop. Hyperplastic lineage scoring was performed manually in Fiji. All cartoon representations were created with BioRender.com (License numbers; Fig.1A: BD25D4AGIN, Fig.S1C: LX25D4C98C, Fig.6A: MW25D4BDTD, Fig.S6 A, D: KW25D4DTZN).

Supplementary Figures

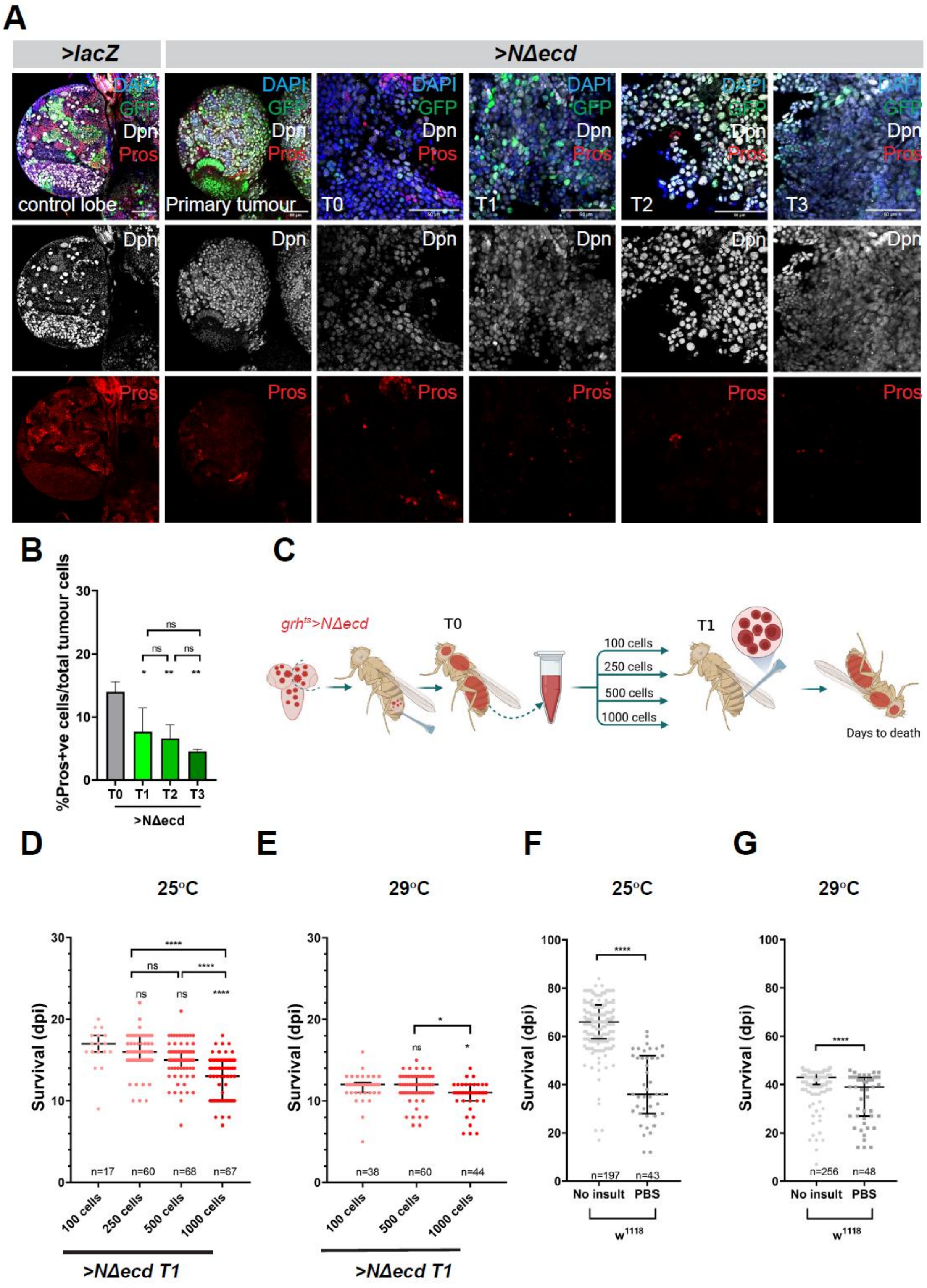

Figure S1: ***NΔecd tumours have lower number of differentiating cells upon serial transplantations and the reduction in host lifespan is proportional to the amount of*** **NΔecd** ***tumourigenic cells injected and temperature dependent.***

(A) Confocal images of GFP marked *act-F/O* clones from control brains (*>lacZ*), primary *>NΔecd* brain hyperplasia (larval brain hemispheres) or *>NΔecd* allograft tumour fragments recovered from the abdomen of host flies (T0 to T3). The tissues are stained for NB marker Dpn (grey) and the early neuronal marker Pros (red). DAPI (blue) shows nuclei. In more advanced allografts (T2 and T3) the number of Pros-positive nuclei is reduced. Scale bar 50µm. **(B)** Proportion of Pros+ve cells over total tumour cells in *NΔecd* allograft tumours at various stages, T0 (N=4), T1(N=4), T2(N=8), T3(N=2). N= the number of tumour explant pieces analysed per stage. Each piece contained 1500 to 3500 tumour cells. **(C)** Schematic representation of grh^{ts}*>NΔecd* hyperplastic larval brains transplantation into adult fly hosts w^{1118} (T0). For T1 allografts 100, 250, 500 or 1000 cells of T0 tumour origin were isolated, counted and injected into new fly hosts. Host survival after transplantation was recorded daily. **(D-E)** Scatter plots of host lifespan after transplantation of 100, 250, 500 and 1000 *>NΔecd* tumour cells. Host flies were kept at 25°C (**D**) or 29°C (**E**). Only tumour-bearing flies were scored. **(F-G)** Scatter plots depicting lifespan of unchallenged (no insult) w^{1118} flies or flies injected with sterile PBS (32nl). Animals were kept at 25°C **(F)** or 29°C **(G).** Note that wild type w^{1118} flies are also susceptible to sterile PBS injection, however with much higher life expectancy compared to the tumour-bearing fly hosts. Middle black line: median values; smaller lower and upper black lines: first to third interquartile ranges; *P_{adj} or $P<0.05$; ** P_{adj} or $P<0.01$; **** P_{adj} or $P<0.0001$, ns: not significant* [ordinary one-way ANOVA, Tukey's multiple comparisons test for (**D, E**); unpaired t-test for (**F, G**)]. n= indicates the total number of flies scored from 3 biologically independent experiments.

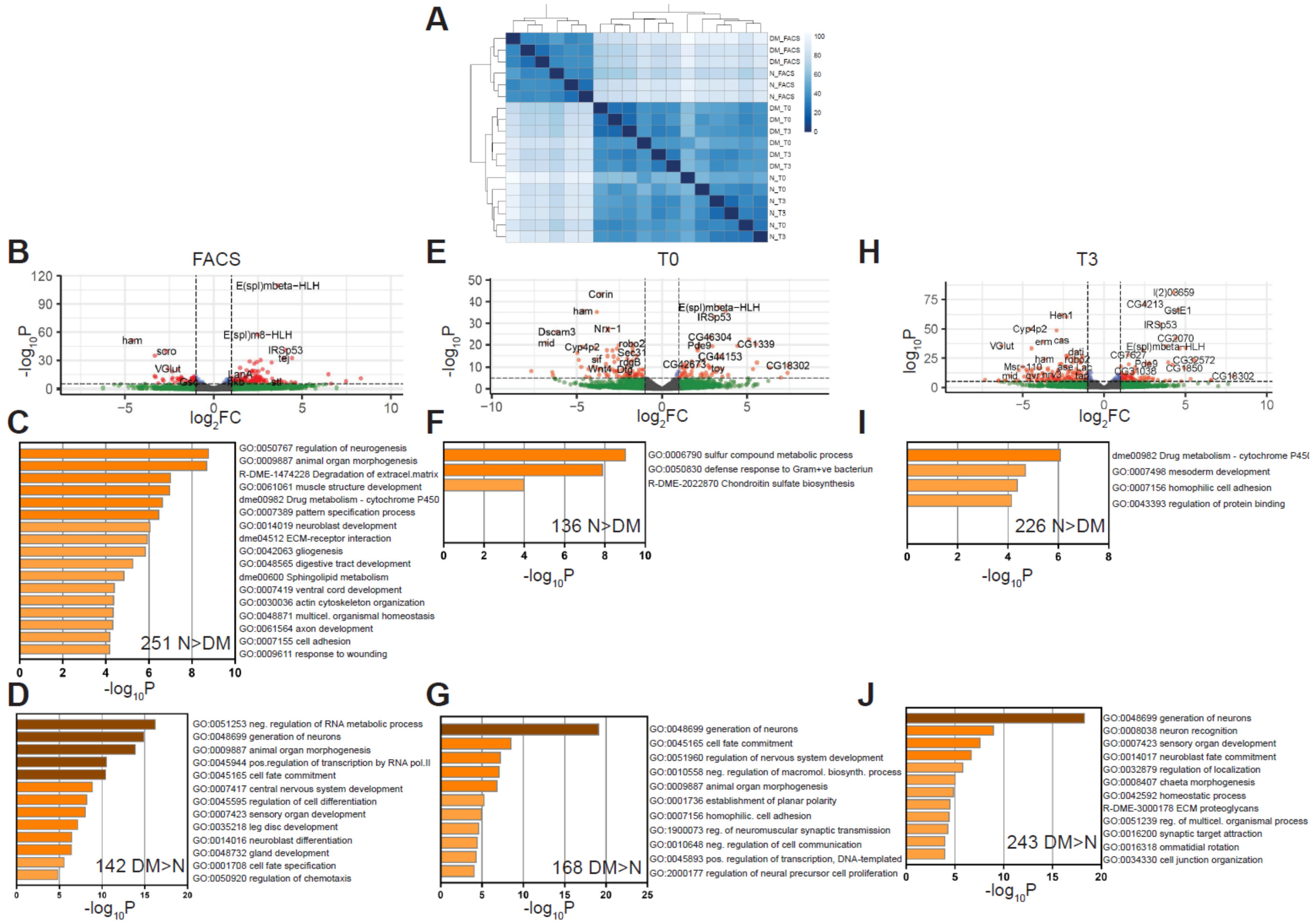

Figure S2: Comparison between NΔecd and DM tumours.

(A) Correlation heatmap with a dendrogram clustering the transcriptomes of *N and DM* primary and allograft tumours. **(B)** Volcano plot comparing *NΔecd* (N) vs *dpn+E(spl)mγ (DM)* FACS sorted primary tumour cells. **(C-D)** Bar graphs of the top enriched GO terms within gene lists of *NΔecd vs DM* upregulated **(C)** and downregulated **(D)** genes in primary tumour cells. **(E)** Volcano plot of *NΔecd vs DM* T0 tumour cells. **(F-G)** Bar graphs of the top enriched GO terms (when present, using enrichment $p_{adj}<0.1$ as a cutoff) within gene lists of *NΔecd vs DM* upregulated **(F)** and downregulated **(G)** genes in T0 tumour cells. **(H)** Volcano plot of *N vs DM* allograft T3 tumour cells. **(I-J)** Bar graphs of the top enriched GO terms within gene lists of *NΔecd vs DM* upregulated **(I)** and downregulated **(J)** genes in T3 tumour cells. Differentially regulated genes were filtered

by FDR≤ 0.05, $|\log_2 FC|$≥0.5, and basemean≥30 and the number of genes in each list is shown in C, D, F, G, I, J.

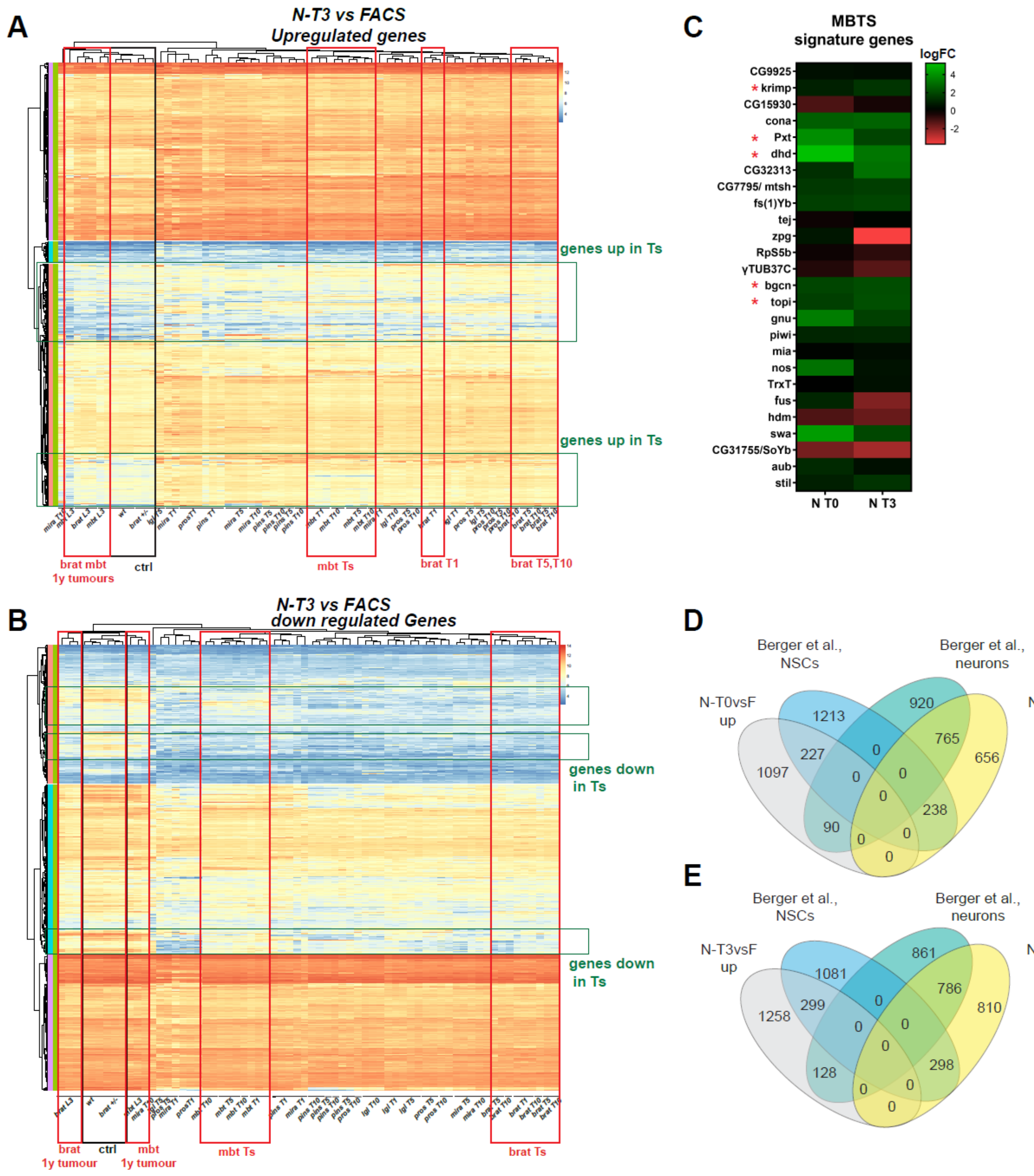

Figure S3: A small number of the differentially expressed genes in NΔecd allograft brain tumours show similar expression trends in other allograft brain tumours and normal NSCs.

(A-B) Heat maps depicting the expression status (microarray signal) of 1165 genes upregulated (**A**) or of 1431 genes downregulated (**B**) in *NΔecd* T3 allograft tumours in various primary and allograft brain tumours generated by mutations in asymmetric cell division genes (*lgl, pins, mira, pros, brat*) or the chromosome architecture factor l*(3)mbt* [20]. Vertical black rectangle delimits control larval brain tissues; vertical red rectangles delimit mutant l*(3)mbt* and *brat* primary (L3) or allograft tumours at the T1, T5 or T10 stage. Horizontal green boxes mark gene clusters that are also upregulated/downregulated in allograft vs L3 l*(3)mbt* and/or *brat* tumours. Note that of the hundreds of genes whose expression is increased (A) or decreased (B) in *NΔecd* allograft tumours, only one third or less changed coordinately in *brat and l(3)mbt* allografts. **(C)** Heat map of the 26 germline *l(3)mbt* signature genes in NΔecd vs primary tumours. Starred genes are upregulated in NΔecd tumours. Note that only 5 germline *l(3)mbt* signature genes are upregulated in NΔecd allograft tumours (**D-E**). Venn diagrams comparing N-T0 and T3 upregulated (**D**) and downregulated (**E**) genes with NSC and neuron enriched gene sets [21]. Note that NΔecd allograft tumours substantially differ from wt NSCs; only a small proportion (T0:17%, T3:19%) of upregulated genes in *NΔecd* tumours overlapped with NSC enriched genes. In contrast, there is a strong overlap (T0: 54%, T3: 49%) between downregulated genes in *NΔecd* allograft tumours and neuron enriched genes, confirming that the former lose neuronal identity.

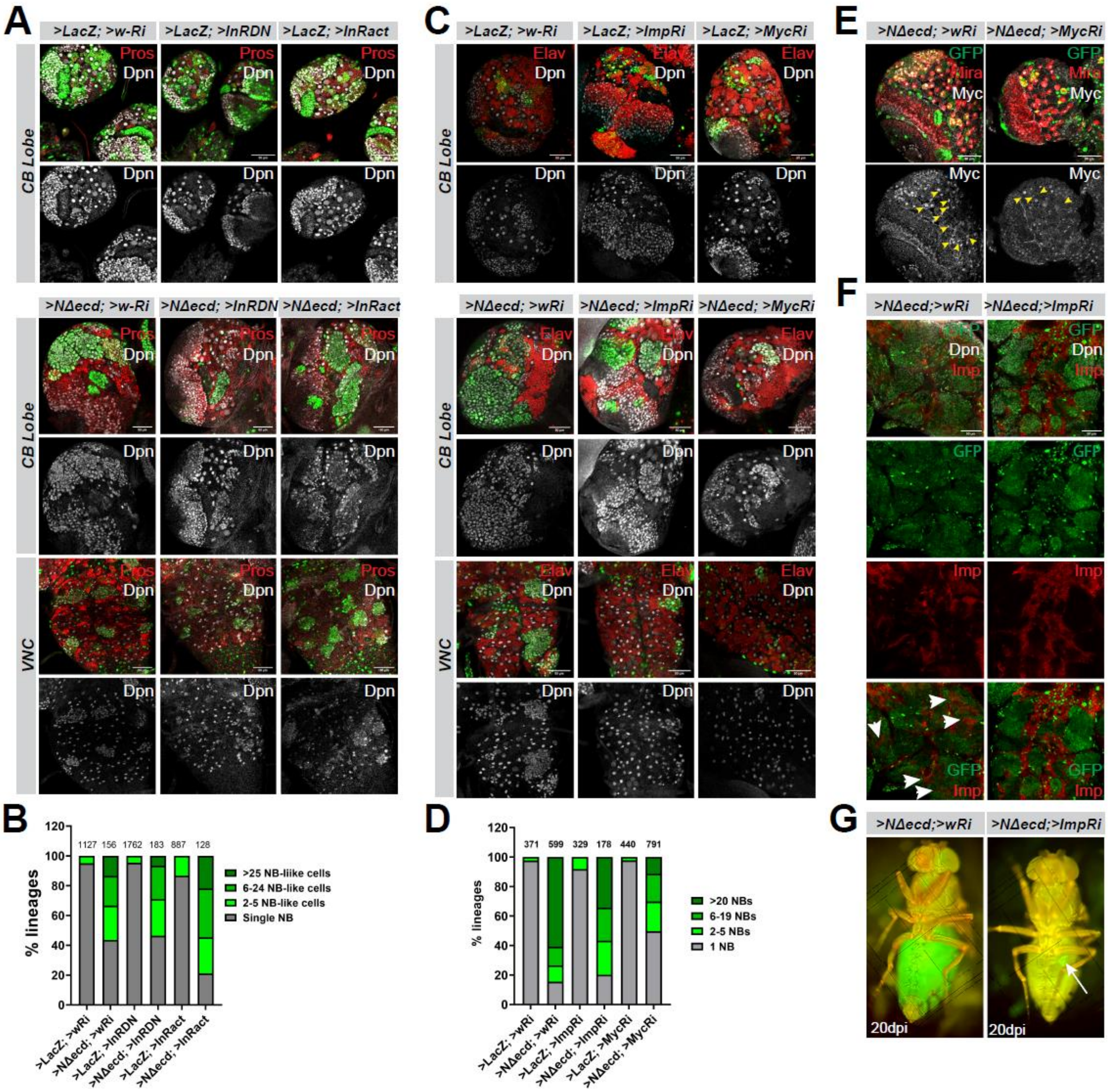

Figure S4. Altering InR signaling or downregulating Imp or Myc affects NΔecd-induced hyperplasia in the larval CNS.

(A) Confocal images of larval brain lobes and VNCs carrying 4 days old *act-F/O* clones of the indicated genotypes [control: *>lacZ; >w-RNAi* or *>lacZ; >InR*^{DN}*(UAS-InR*^{K1409A}*)* or *>lacZ; >InR*^{act}*(UAS-InR*^{A1325D}*)* and hyperplastic*: >NΔecd; >w-RNAi* or *>NΔecd; >InR*^{DN} or *>NΔecd; >InR*^{act}*]* stained for Dpn (grey) and the GMC/early neuronal marker Pros (red). Scale bar 50μm. Note that hyperplastic GFP-positive lineages in *>NΔecd; >InRDN* are smaller in size and contain fewer Dpn-positive cells than *>NΔecd;>w-RNAi*. **(B)** Diagram depicting the frequency of NB

lineages with one (single NB) or multiple Dpn-positive cells (2-5, 6-24, >25 Dpn-positive NB-like cells) for the indicated genotypes. The total number of clones scored per genotype from 3 biologically independent experiments is shown above each bar. Type II lineages, that are highly proliferative [2], [24]–[26], were excluded from this analysis. **(C)** Confocal images of larval brain lobes and VNCs with GFP-marked (green) 4 day old *act-F/O* clones of the indicated genotypes stained for Dpn (grey) and the neuronal marker Elav (red). Scale bar 50µm. Note that *>NΔecd; >Imp-RNAi* and *NΔecd;>Myc-RNAi* lineages are smaller in size and contain fewer Dpn-positive cells than *>NΔecd; >w-RNAi* (control). **(D)** Diagram depicting the frequency of NB lineages with one or multiple NBs (2-5, 6-19, >20 Dpn-positive NB-like cells) for the indicated genotypes. The total number of clones scored per genotype from 3 biologically independent experiments is shown above each bar. Type II lineages, that are highly proliferative, were excluded from this analysis. Note that wt lineages occasionally contain two Dpn-positive cells, as early GMCs have not downregulated Dpn yet [27]. We did not observe any NB loss upon the *Myc RNAi* with the *actF/O* driver, however we did notice that the NBs often became smaller, in agreement with [28]. **(E-F)** Confocal images of larval brain lobes **(E)** and VNCs **(F)** with GFP-marked (green) 4 day old *act-F/O* clones of the indicated genotypes stained for Myc (grey; E) and Mira (red; E) or Dpn (grey; F) and Imp (red; F). Yellow arrowheads point to *>NΔecd; >w-RNAi or NΔecd;>Myc-RNAi* clones, where Myc is silenced in the latter. White arrows in **(F)** point to *>NΔecd; >w-RNAi* clones with some Imp+ve cancer NSCs. Note that Imp is completely abolished from all *>NΔecd; >Imp-RNAi* clones. Scale bar 50µm **(G)** Host flies carrying *>NΔecd; >w-RNAi* and *>NΔecd; >Imp-RNAi* allografts at 20 days post injection (dpi). Whereas the control tumour has colonized the entire host (left), only a small fragment of surviving Imp-RNAi tumour is seen (arrow).

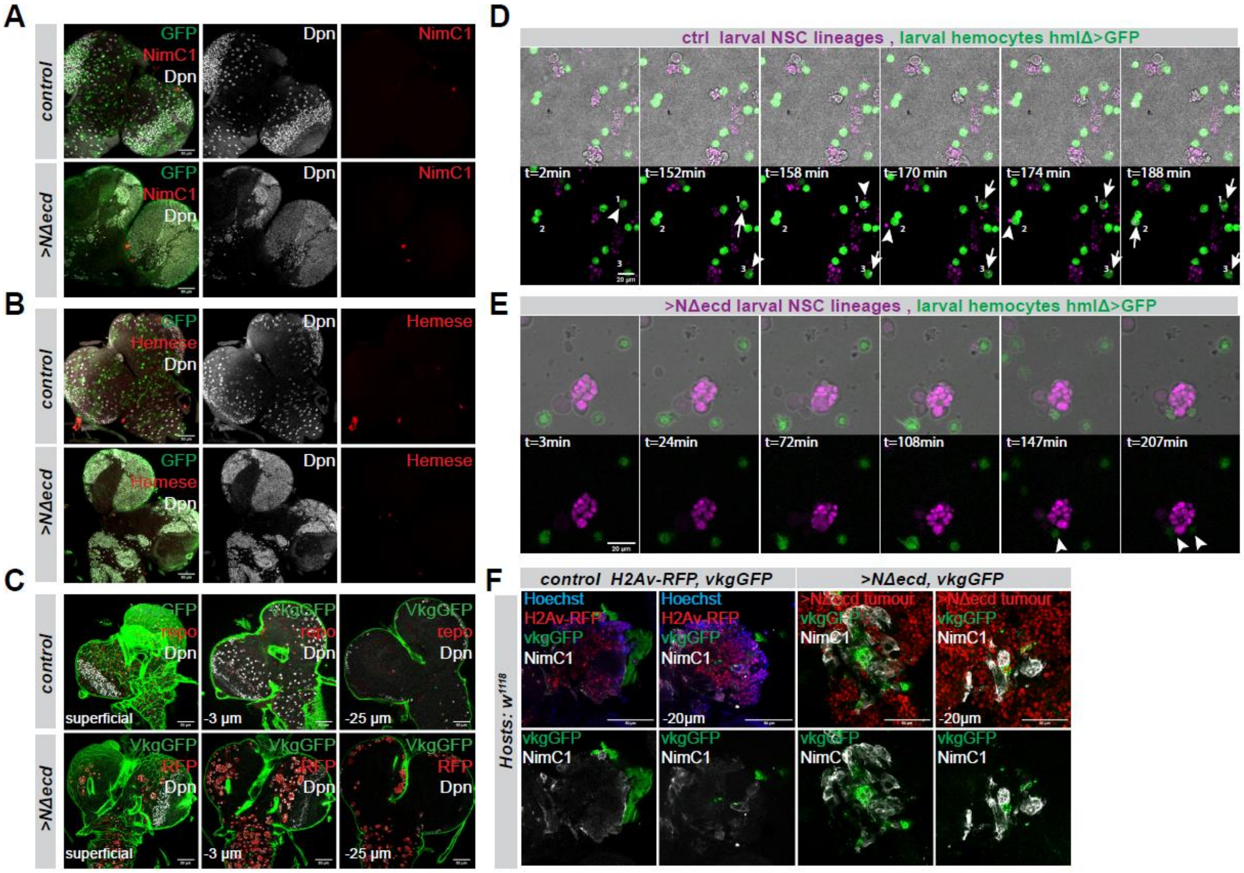

Figure S5: Larval haemocytes are not attracted by primary NΔecd tumours in intact larval CNSs but are attracted to dissociated neuroblast lineages.

(A-B) Confocal images of larval brain lobes with *act-F/O >lacZ* (control) or *>NΔecd* stained for Dpn (grey) and two haemocyte markers, NimC1 **(A)** or Hemese **(B)**. Note that few to no haemocytes are attached to the CNS of either genotype. Scale bar 50µm. **(C)** Confocal images of control and grh^{ts} *> NΔecd+RedStinger* hyperplastic larval brain lobes. Dpn (grey) stains NSC-like cells and RFP labels *grh*-expressing cells. vkg-GFP marks the basement membrane, which is not disrupted by the *NΔecd* induced hyperplasia. Repo marks glial cell nuclei. Scale bar 50µm. **(D, E)** Time points from a time-lapse movie of wt (ubiquitous *H2Av-RFP*) **(D)** or grh^{ts}*> NΔecd+RedStinger* **(E)** NB lineages from dissociated larval CNS (magenta) co-cultured ex vivo with larval haemocytes marked by *hmlΔ>GFP* (green). Top: fluorescent image superimposed on

brightfield. Arrowheads point to brain cells contacting haemocytes while arrows point to engulfment events. Haemocytes can capture and phagocytose both wt and tumourous NB lineages when the glial blood brain barrier is absent. Scale bar 20μm**.** **(F)** Confocal images of vkg-GFP marked wt (ubiquitous *H2Av-RFP*) or *grh*^{ts}*> NΔecd+RedStinger* explanted brain tissue a few days post transplantation into *w*^{1118} hosts stained for NimC1(grey). Note that in both cases the basement membrane is shed and multiple haemocytes attach throughout the explants.

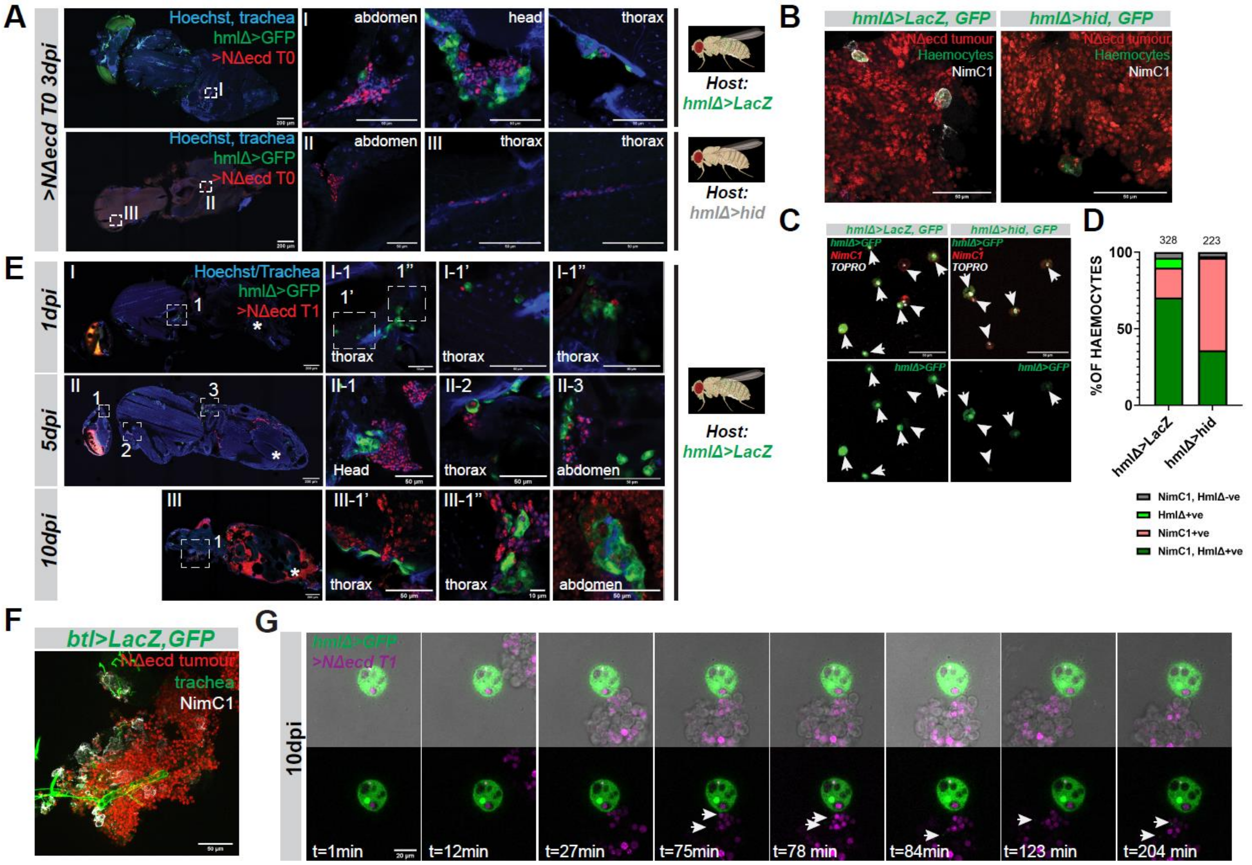

Figure S6: Adult haemocytes associate with tumour and tracheae in whole fly sections and extend protrusions to attach to tumour

(A) Confocal images of cryosections from whole adult fly hosts, *hmlΔ>GFP+ lacZ* vs *hmlΔ*>GFP +*hid*, transplanted with grh^{ts} *>NΔecd+RedStinger* brain lobes (red) 3 days post transplantation. Note the near complete absence of haemocytes (green) from *hmlΔ>GFP +hid* hosts. Hoechst stains nuclei. Trachea tubes and airsacs are autofluorescent upon 405nm laser excitation (blue). Panels marked I-III are enlargements of the boxed regions in the whole-fly images. The remaining enlargements come from different fly sections. **(B)** Confocal images of grh^{ts} *>NΔecd+RedStinger* T0 explants grown in *hmlΔ>GFP+ lacZ* or *hmlΔ>GFP +hid* hosts. Tumour pieces are stained with the haemocyte marker NimC1 (white). Very few haemocytes remain on the *NΔecd* allograft extracted from *hmlΔ>GFP, hid* hosts. **(C)** Confocal images of haemocytes upon bleeding of

hmlΔ>GFP+ lacZ or *hmlΔ>GFP +hid* adult flies 6 days after eclosion. NimC1 (red) stains haemocytes and TOPRO stains nuclei. Arrows: hmlΔ>GFP positive haemocytes, arrowheads: NimC1+ haemocytes with reduced or no expression of hmlΔ>GFP. There is a great reduction in GFP-positive cells upon *hid* expression, and almost all remaining haemocytes are NimC1-positive. **(D)** Graph depicting the proportions of various haemocyte subpopulations present upon bleeding from adult *hmlΔ>lacZ+GFP* control (15 flies) and *hmlΔ>hid+GFP* (30 flies) flies. 328 cells were recovered and stained for NimC1 from the former and 223 cells from the latter. Note that after *hmlΔ>hid* treatment, the GFP-positive (Hml-positive) population drops from 70% to 35%, as expected because of the ablation. The NimC1-positive population increases from 90% to 96%. The proportion of each subpopulation is calculated by dividing over total cells recovered (TOPRO). **(E)** A time course of whole adult cryosections from *hml>GFP +lacZ* hosts bearing *NΔecd* allograft tumours (red; T1 stage) at 1, 5 and 10 days' post injection (dpi) of 500 T0 cells. Hoechst marks nuclei and trachea autofluorescence. Note that tumour cells are often found in close proximity to haemocytes and tracheae and that haemocytes often carry vacuoles with tumour cell material. Scale bar 200 μm and 50 μm in closeups. Closeup panels correspond to the boxed sections of the wide view, as marked. III-1' and III-1" are the same region at two different focal planes. The unmarked closeup comes from a different fly section. **(F)** Confocal image of a grh^{ts} *>NΔecd+RedStinger* T0 explant grown in a *btl>GFP* host, which marks all tracheae. Note NimC1-positive haemocytes (grey) associating with tracheal tubes within the tumour fragment. Scale bar 50μm. **(G)** Stills from a timelapse movie of grh^{ts}*>NΔecd* allograft tumour cells (magenta) raised in *hml>GFP+ lacZ* host (green haemocytes) for 10 days prior to explanting and imaging at the T1 stage. Top: fluorescent image superimposed on brightfield. Note the halo of GFP-positive haemocyte filopodia which extend around the haemocyte. A big tumour clump is trapped when it

gets close to the haemocyte at t=12min. Haemocyte filopodia remain attached to the tumour clump after capture (arrows). Arrows indicate GFP-positive filopodia. Scale bar 20µm.

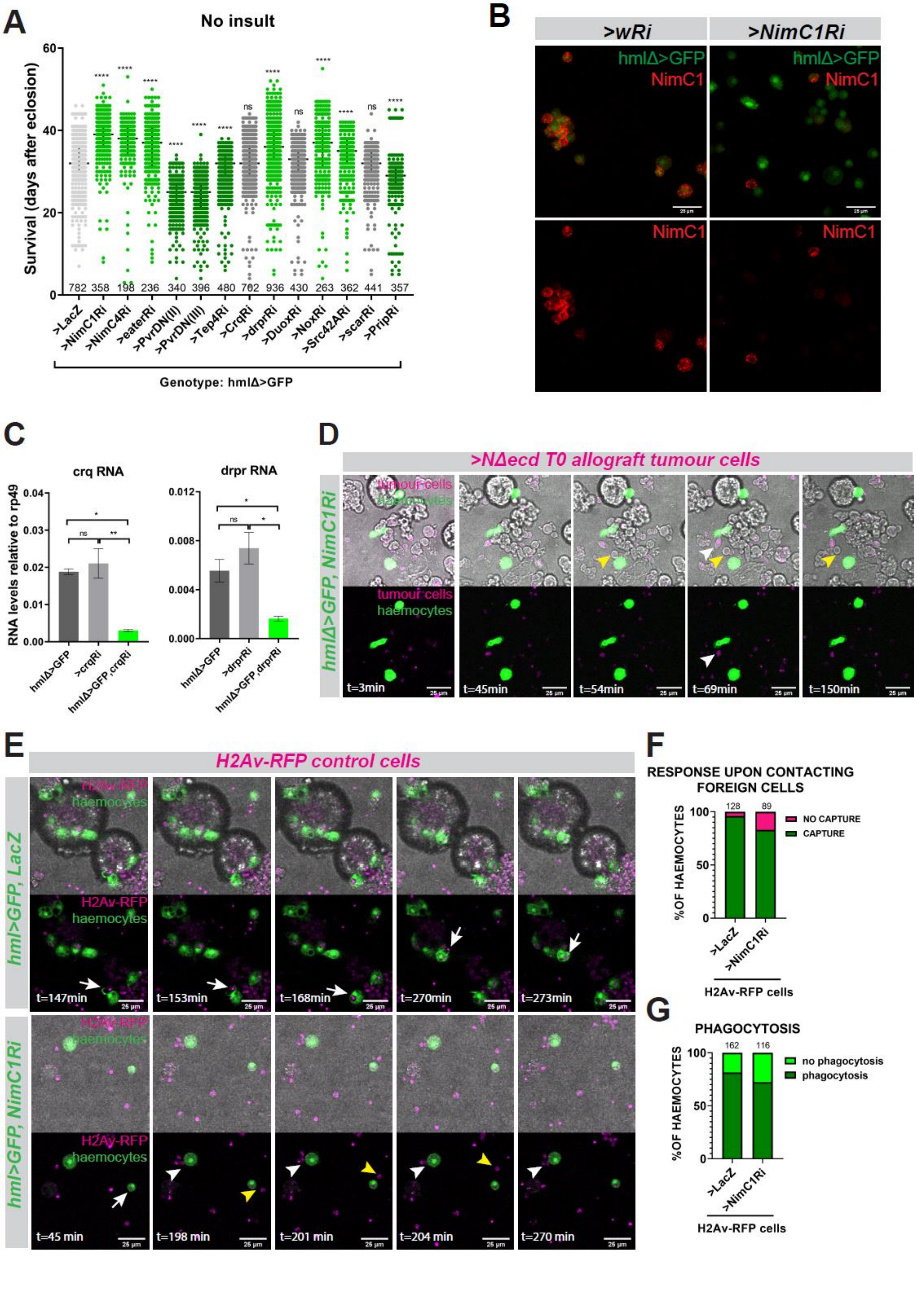
A
No insult
Survival (days after eclosion)
782 358 198 236 340 396 480 702 936 430 263 362 441 357
>LacZ
>NimC1Ri
>NimC4Ri
>eaterRi
>PvrDN(II)
>PvrDN(III)
>Tep4Ri
>CrqRi
>drprRi
>DuoxRi
>NoxRi
>Src42ARi
>scarRi
>PripRi
Genotype: hmlΔ>GFP
B
>wRi
>NimC1Ri
hmlΔ>GFP
NimC1
C
crq RNA
drpr RNA
RNA levels relative to rp49
hmlΔ>GFP
>crqRi
hmlΔ>GFP,crqRi
>drprRi
hmlΔ>GFP,drprRi
D
>NΔecd T0 allograft tumour cells
hmlΔ>GFP, NimC1Ri
tumour cells
haemocytes
t=3min
t=45min
t=54min
t=69min
t=150min
E
H2Av-RFP control cells
hml>GFP, LacZ
hml>GFP, NimC1Ri
H2Av-RFP
haemocytes
t=147min
t=153min
t=168min
t=270min
t=273min
t=45 min
t=198 min
t=201 min
t=204 min
t=270 min
F
RESPONSE UPON CONTACTING FOREIGN CELLS
%OF HAEMOCYTES
128 89
NO CAPTURE
CAPTURE
>LacZ
>NimC1Ri
H2Av-RFP cells
G
PHAGOCYTOSIS
%OF HAEMOCYTES
162 116
no phagocytosis
phagocytosis
>LacZ
>NimC1Ri
H2Av-RFP cells

Figure S7: Effect of NimC1 and other phagocytic receptors knockdown on lifespan and tumour capture.

(A) Scatter plot that depicts the lifespan of unchallenged hosts of the indicated genotypes (*hmlΔ>RNAi* for the genes tested in the screen, see fig.6). Middle black lines: median values; lower and upper black lines: first to third interquartile ranges; ****P_{adj}<0.0001, ns: not significant (ordinary one-way ANOVA, Dunnett's multiple comparisons test). n= indicates the total sample size from all 3 independent biological experiments. Note that knockdown of many genes in haemocytes, including phagocytic receptors *NimC1*, *eater* and *NimC4*, has a positive effect on lifespan (light green box plots). Genes whose knockdown resulted in lifespan reduction are marked with dark green box plots. Genes that showed no effect are marked in grey. Flies were kept at 29°C. **(B)**Efficiency of NimC1 knockdown. Larval L3 haemocytes of the indicated genotypes were isolated and stained for NimC1. The majority of *hml*-positive haemocytes has lost NimC1 upon RNAi. Scale bar 25 µm. **(C)** Fold change in RNA levels of crq and drpr in larval haemocytes bled from 10 larvae of the indicated genotypes: *hmlΔ>GFP, >crq-RNAi only, hmlΔ>GFP, crq-RNAi* (left) and *hmlΔ>GFP, >drpr-RNAi only, hmlΔ>GFP, drpr-RNAi* (right). **(D)** NimC1 compromised adult haemocytes cannot efficiently capture *grh*^{ts}*>NΔecd* tumour cells. Time points from a timelapse movie of *grh*^{ts}*>NΔecd* allograft tumour cells (magenta) interacting with *hmlΔ>GFP+NimC1-RNAi* haemocytes (green). Two tumour cells are highlighted by arrowheads. One (white arrowhead) is captured by a haemocyte at t= 69 min. The other (yellow arrowhead) makes an initial contact with another haemocyte at t= 54 min, but subsequently bounces away, indicating unsuccessful entrapment. The large non-fluorescent cells seen in these images are probably fat-body cells co-isolated with the tumour. Scale bar 25 µm. **(E)** Stills from timelapse movies of control (H2Av-RFP) brain cells (magenta) co-cultured with naïve isolated adult

haemocytes (green) from *hmlΔ>GFP+lacZ* (control) and *hmlΔ>GFP+NimC1-RNAi* flies. White arrows indicate control cells being engulfed by haemocytes, white arrowheads point to trapped cells but not phagocytosed whereas yellow arrowheads point to instances of approaching control cells with no capture by haeomocytes. Scale bar 25μm. **(F-G)** Diagram depicting the proportion of haemocytes that capture control cells upon contact (**F**) or that contain cells or cellular debris in their phagosomes after 4 hours of coculture (**G**). The number of total contact events scored is shown above each bar.

Materials

REAGENT or RESOURCE	SOURCE	IDENTIFIER
Antibodies		
guinea pig anti-Dpn (1:2000)	Delidakis C. lab; [6]	N/A
mouse anti-Pros (1:50)	Developmental Studies Hybridoma Bank, DSHB	DSHB Cat# Prospero (MR1A), RRID:AB_528440
rabbit anti-GFP (1:10000)	Minotech	Cat# 701-1
rat anti-Elav (1:300)	Developmental Studies Hybridoma Bank, DSHB	Cat# Rat-Elav-7E8A10; RRID:AB_528218
mouse anti-Miranda (1:100)	Fumio Matsuzaki, Kobe, Japan [29]	N/A
mouse anti-NimC1 (P1 antigen) (1:30)	Ando I. lab; [30]	N/A
mouse anti-Hemese (1:100)	Ando I. lab; [31]	N/A
rabbit anti-PH3 (1:100)	Millipore	Millipore Cat# 06-570, RRID:AB_310177
mouse anti-GFP (1:20)	Developmental Studies Hybridoma Bank, DSHB	DSHB Cat# GFP-G1, RRID:AB_2619561
guinea pig anti-Myc (1:100)	Claude Desplan	N/A
rat-anti-Imp (1:100)	Claude Desplan	N/A
Alexa Fluor ® 488 Goat anti-Rabbit IgG	Thermo Fisher Scientific	Thermo Fisher Scientific Cat# A-11034, RRID:AB_2576217
Alexa Fluor ® 488 Goat anti-Mouse IgG	Molecular Probes	Molecular Probes Cat# A-11029, RRID:AB_2534088
Alexa Fluor ® 555 Goat anti-Mouse IgG	Molecular Probes	Molecular Probes Cat# A-21424, RRID:AB_141780
Cy3-AffiniPure F(ab')2 Frag Donkey Anti-rat IgG	Jackson ImmunoResearch Labs	Jackson ImmunoResearch Labs Cat# 712-166-153, RRID:AB_2340669
Alexa Fluor ® 555 Donkey Anti-Rabbit IgG	Molecular Probes	Molecular Probes Cat# A-31572, RRID:AB_162543
Alexa Fluor ® 647 Goat Anti-Guinea Pig IgG	Molecular Probes	Molecular Probes Cat# A-21450, RRID:AB_141882

Alexa Fluor ® 633 Goat Anti-Mouse IgG	Molecular Probes	Molecular Probes Cat# A-21052, RRID:AB_2535719
**Chemicals, peptides, and recombinant proteins**		
Trizol	Invitrogen now ThermoFischer Scientific	Cat# 15596026
Tissue-Tek® O.C.T. Compound	SAKURA	Cat# 4583
Formaldehyde, 10%, methanol free, Ultra-Pure	Polysciences Europe GmbH	Cat# 04018-1
Triton® X-100	Merck	Cat# 1086431000
Albumin (BSA) Fraction V	PanReac Applichem, ITW reagents	Cat# A1391.0100
Schneider's Drosophila medium	Gibco™	Cat# 21720024
D-(+)-Glucose monohydrate	Sigma-Aldrich	Cat# 16301
Fetal Bovine Serum	Gibco™	Cat# 10270106
Insulin solution human	Sigma-Aldrich	Cat# I9278
Antibiotic-Antimycotic (100X)	Thermo Fisher Scientific	Cat# 15240062
Collagenase from *Clostridium histolyticum*	Sigma-Aldrich	Cat# C0130
2′,7′-Dichlorofluorescin diacetate	Sigma-Aldrich	Cat# D6883
Sucrose	Sigma-Aldrich	Cat# 84100
Propyl gallate	Sigma-Aldrich	Cat# 02370-100G
Glycerol anhydrous	Applichem GmbH	Cat# A2364,1000
Drop-n-Stain EverBrite™ Mounting Medium	Biotium	Cat# 23009
DAPI	PAnReac Applichem Reagents	Cat# A4099,0005
Hoechst 33258	Sigma-Aldrich	Cat#: 94403-1ML
**Critical commercial assays**		
Luciferase Assay System	Promega	Cat# E1500
polyA mRNA magnetic isolation kit (NEB)	New England Biolabs	Cat# E7490L
the NEB Ultra II RNA library kit for Illumina	New England Biolabs	Cat# E7770L
NextSeq™ 500/550 High Output Kit v2.5 (150 cycles)	Illumina	Cat# 20024907
**Deposited data**		
[20]	Microarray Data	Sup. Material info Table S2
[2]	ChIP data	GSE68614
[6]	ChIP data	GSE141794
[5]	RNA seq data	GSE179507
**Experimental models: Organisms/strains**		
D.melanogaster w^{1118}	Bloomington Drosophila Stock Center	RRID: BDSC_5905

D.melanogaster y w^{67c23}	Bloomington Drosophila Stock Center	RRID: BDSC_6599
D.melanogaster hsFLP;;act>STOP>Gal4, UAS-nlsGFP	[6]	N/A
tubP -Gal80^{ts}*,UAS-RedStinger/Cyotb; grhNB-Gal4/TM6B*	[2]	N/A
D.melanogaster yw; UAS-NΔecd	[1]	N/A
D.melanogaster UAS-lacZ	Bloomington Drosophila Stock Center	RRID: BDSC_3956
D.melanogaster UAS-lacZ	Bloomington Drosophila Stock Center	RRID: BDSC_3955
D.melanogaster UAS-w-RNAi	Bloomington Drosophila Stock Center	RRID: BDSC_35573
UAS-lacZ; UAS-wRi	This study	N/A
UAS-NΔecd; UAS-wRi	This study	N/A
D.melanogaster UAS-InR DN	Bloomington Drosophila Stock Center	RRID:BDSC_8253
UAS-lacZ; UAS-InRDN	This study	N/A
UAS-NΔecd; UAS-InRDN	This study	N/A
D.melanogaster UAS-InR act	Bloomington Drosophila Stock Center	RRID:BDSC_8440
UAS-lacZ; UAS-InRact	This study	N/A
UAS-NΔecd; UAS-InRact	This study	N/A
D.melanogaster UAS-MycRi	Bloomington Drosophila Stock Center	RRID:BDSC_36123
UAS-lacZ; UAS-MycRi	This study	N/A
UAS-NΔecd; UAS-MycRi	This study	N/A
D.melanogaster UAS-ImpRi	Bloomington Drosophila Stock Center	RRID:BDSC_34977
UAS-lacZ; UAS-ImpRi	This study	N/A
UAS-NΔecd; UAS-ImpRi	This study	N/A
D.melanogaster hmlΔGal4,UAS-2XEGFP	Bloomington Drosophila Stock Center	RRID:BDSC_30140
D.melanogaster UASluciferase	Bloomington Drosophila Stock Center	RRID:BDSC_35788
UAS-NΔecd/Cyotb; UAS-luciferase/TM6B	This study	N/A
UAS-lacZ/Cyotb; UAS-luciferase/TM6B	This study	N/A
D.melanogaster UAS-hid/Cyotb	Bloomington Drosophila Stock Center	RRID:BDSC_65403
D.melanogaster UAS-NimC1-Ri , validated by [32]	Bloomington Drosophila Stock Center	RRID:BDSC_25787
D.melanogaster UAS-NimC4-Ri	Bloomington Drosophila Stock Center	RRID:BDSC_61866
D.melanogaster UAS-eater-Ri, validated by [32]	Bloomington Drosophila Stock Center	RRID:BDSC_25863
D.melanogaster UAS-Tep4 RNAi	Bloomington Drosophila Stock Center	RRID:BDSC_67218
D.melanogaster UAS-Pvr DN	Bloomington Drosophila Stock Center	RRID:BDSC_58431

D.melanogaster UAS-Pvr DN/Cyo	Bloomington Drosophila Stock Center	RRID:BDSC_58430
D.melanogaster UAS-crq-Ri	Bloomington Drosophila Stock Center	RRID:BDSC_40831
D.melanogaster UAS-draper-Ri, validated by [33]	Bloomington Drosophila Stock Center	RRID:BDSC_36732
D.melanogaster UAS-Prip-Ri	Bloomington Drosophila Stock Center	RRID:BDSC_44464
D.melanogaster UAS-Duox-Ri, validated by [33]	Bloomington Drosophila Stock Center	RRID:BDSC_33975
D.melanogaster UAS-Nox-Ri	Bloomington Drosophila Stock Center	RRID:BDSC_32902
D.melanogaster UAS-Scar-Ri	Bloomington Drosophila Stock Center	RRID:BDSC_51803
D.melanogaster UAS-src4aARi	Bloomington Drosophila Stock Center	RRID:BDSC_55868
Sp/CyO;srp-moeisin-3xmCherry	Siekhaus lab, [34]	N/A
D.melanogaster Vkg-GFP	KYOTO Drosophila Stock Center	RRID:DGGR_110692
D.melanogaster His2Av-RFP	Bloomington Drosophila Stock Center	RRID:BDSC_23651
Software and algorithms		
ImageJ (Fiji)	NIH	RRID:SCR_003070 https://imagej.nih.gov/ij/docs/guide/146-2.html
GraphPad Prism (8.0)	GraphPad Software Inc., La Jolla, CA, USA	RRID:SCR_002798 https://www.graphpad.com/scientific-software/prism/
Adobe Photoshop (CC)	Adobe	RRID:SCR_014199 https://www.adobe.com/products/photoshop.html
Adobe Illustrator (CC)	Adobe	RRID:SCR_010279 https://www.adobe.com/products/illustrator.html
StarDist	[35]	https://imagej.net/plugins/stardist
R (v4.0.2)with DEseq2 (v1.30.1) package	RStudio	https://genomebiology.biomedcentral.com/articles/10.1186/s13059-014-0550-8
FastQC (v0.11.9)	Linux	https://www.bioinformatics.babraham.ac.uk/projects/fastqc/

STAR (v2.7.5)	Linux	https://www.ncbi.nlm.nih.gov/pmc/articles/PMC3530905/
featureCounts (v2.0.1)	Linux	https://pubmed.ncbi.nlm.nih.gov/24227677/
Leica Application Suite X (LAS X)	Leica Microsystems	RRID:SCR_013673<br><br>https://www.leica-microsystems.com/products/microscope-software/p/leica-las-x-ls/
BioRender	BioRender	RRID:SCR_018361 https://biorender.com/
Other		
Nanoject II Auto-Nanoliter Injector	Drummond Scientific Company	Cat# 3-000-205A
PC-10 Puller	Narishige International	N/A
Stereoscope	Leica Microsystems	MZ FLIII stereoscope
Confocal microscope	Leica Microsystems	TCS-SP8
pE-300white Illumination System - Single Band	CoolLED	Cat# pE-300-W-D-SB-31E-20
Luminometer	Turner Designs	Cat# TD-20210
Leica Cryostat	Leica Biosystems	Cat# CM1850
Mattek 35mm dish	Mattek	Cat#P35G-1.5-14-C
Falcon® 5 mL Round Bottom Polystyrene Test Tube, with Cell Strainer Snap Cap	Corning	Cat# 352235
Epredia™ SuperFrost Plus™ Adhesion slides	Fischer Scientific	Cat# J1800AMNZ

Dataset Legends

Dataset S1: Transcriptome analysis of allograft T0 vs primary (FACS) NΔecd tumours, T3 vs primary (FACS) and T3 vs T0. RNA-seq Differential Analysis complete output by DESeq2 analysis**.** *NA* values indicate that for the specific gene, either all samples have zero counts or a single sample contains an extreme count outlier, as described in DESeq2 R package**.**

Dataset S2: Metascape analysis of differentially expressed gene lists from allografted NΔecd tumours compared to the primary (FACS) NΔecd tumour. Input genes were filtered for $|\log_2 FC| \geq 0.5$, padj ≤ 0.05, base mean ≥ 30.

Dataset S3: Transcriptome analysis of NΔecd vs DM primary (FACS) tumours, NΔecd vs DM T0 tumours and NΔecd vs DM T3 tumours. RNA-seq Differential Analysis complete

output by DESeq2 analysis**.** *NA* values indicate that for the specific gene, either all samples have zero counts or a single sample contains an extreme count outlier, as described in DESeq2 R package.

Dataset S4: Metascape analysis of DEG lists from allografted or primary NΔecd tumours compared to DM tumours of the same stage. Input genes were filtered for $|log_2FC| \geq 0.5$, $p_{adj} \leq 0.05$, base mean ≥30.

Movie Legends

Movie S1: *grh^{ts}>NΔecd* tumour cells (magenta) raised in an *hml>GFP+lacZ* host (green haemocytes) for 10 days prior to explanting and imaging live. Timelapses were captured every 2min for a duration of circa 4 hours. Top: fluorescent image superimposed on brightfield to visualize cell morphology. Scale bar 20µm. Movie is related to Fig.5E.

Movie S2: *grh^{ts}>NΔecd* allograft tumour cells (magenta) co-cultured with naïve isolated adult haemocytes (green) from *hmlΔ>GFP+lacZ* (control). Timelapses were captured every 3min for a duration of circa 4-6 hours. Top: fluorescent image superimposed on brightfield to visualize cell morphology. Note the highly motile filopodia emanating from the haemocytes. Scale bar 25µm. Movie is related to Fig.7A.

Movie S3: *grh^{ts}>NΔecd* allograft tumour cells (magenta) co-cultured with naïve isolated adult haemocytes (green) from *hmlΔ>GFP+NimC1-RNAi*. Timelapses were captured every 3min for a duration of circa 4-6 hours. Top: fluorescent image superimposed on brightfield to visualize cell morphology. Scale bar 25µm. Movie is related to Fig.7B.

References

[1] S. Fuerstenberg and E. Giniger, “Multiple roles for Notch in Drosophila myogenesis,” *Dev Biol*, 1998, doi: 10.1006/dbio.1998.8944.

[2] E. Zacharioudaki, B. E. Housden, G. Garinis, R. Stojnic, C. Delidakis, and S. Bray, “Genes implicated in stem-cell identity and temporal-program are directly targeted by Notch in neuroblast tumours,” *Development*, Jan. 2015, doi: 10.1242/dev.126326.

[3] A. GOTO *et al.*, “A Drosophila haemocyte-specific protein, hemolectin, similar to human von Willebrand factor,” *Biochemical Journal*, vol. 359, no. 1, p. 99, Oct. 2001, doi: 10.1042/0264-6021:3590099.

[4] F. Rossi and C. Gonzalez, “Studying tumor growth in Drosophila using the tissue allograft method,” *Nat Protoc*, vol. 10, no. 10, pp. 1525–1534, Oct. 2015, doi: 10.1038/nprot.2015.096.

[5] C. Voutyraki *et al.*, “Repression of differentiation genes by Hes transcription factors fuels neural tumour growth in *Drosophila*,” *Int J Dev Biol*, vol. 66, no. 1-2–3, pp. 211–222, 2022, doi: 10.1387/ijdb.210187cd.

[6] S. S. Magadi *et al.*, “Dissecting Hes-centred transcriptional networks in neural stem cell maintenance and tumorigenesis in *Drosophila*,” *Development*, vol. 147, no. 22, Nov. 2020, doi: 10.1242/dev.191544.

[7] S. Gong *et al.*, “Tumor Allotransplantation in Drosophila melanogaster with a Programmable Auto-Nanoliter Injector,” *Journal of Visualized Experiments*, no. 168, Feb. 2021, doi: 10.3791/62229.

[8] P. Sanchez Bosch *et al.*, “Adult Drosophila Lack Hematopoiesis but Rely on a Blood Cell Reservoir at the Respiratory Epithelia to Relay Infection Signals to Surrounding Tissues,” *Dev Cell*, vol. 51, no. 6, pp. 787-803.e5, Dec. 2019, doi: 10.1016/j.devcel.2019.10.017.

[9] A. Daskalaki *et al.*, “Distinct intracellular motifs of Delta mediate its ubiquitylation and activation by Mindbomb1 and Neuralized,” *Journal of Cell Biology*, vol. 195, no. 6, pp. 1017–1031, Dec. 2011, doi: 10.1083/jcb.201105166.

[10] C. J. Evans, T. Liu, and U. Banerjee, “Drosophila hematopoiesis: Markers and methods for molecular genetic analysis,” *Methods*, vol. 68, no. 1, pp. 242–251, Jun. 2014, doi: 10.1016/j.ymeth.2014.02.038.

[11] J. Adams, A. Casali, and K. Campbell, “Sensitive high-throughput assays for tumour burden reveal the response of a drosophila melanogaster model of colorectal cancer to standard chemotherapies,” *Int J Mol Sci*, vol. 22, no. 10, 2021, doi: 10.3390/ijms22105101.

[12] M. Markstein, S. Dettorre, J. Cho, R. A. Neumüller, S. Craig-Müller, and N. Perrimon, “Systematic screen of chemotherapeutics in Drosophila stem cell tumors,” *Proc Natl Acad Sci U S A*, vol. 111, no. 12, 2014, doi: 10.1073/pnas.1401160111.

[13] H. Harzer, C. Berger, R. Conder, G. Schmauss, and J. A. Knoblich, “FACS purification of Drosophila larval neuroblasts for next-generation sequencing,” *Nat Protoc*, vol. 8, no. 6, pp. 1088–1099, Jun. 2013, doi: 10.1038/nprot.2013.062.

[14] S. Andrews, “FASTQC. A quality control tool for high throughput sequence data,” 2010.

[15] A. Dobin *et al.*, “STAR: ultrafast universal RNA-seq aligner,” *Bioinformatics*, vol. 29, no. 1, pp. 15–21, Jan. 2013, doi: 10.1093/bioinformatics/bts635.

[16] Y. Liao, G. K. Smyth, and W. Shi, “featureCounts: an efficient general purpose program for assigning sequence reads to genomic features,” *Bioinformatics*, vol. 30, no. 7, pp. 923–930, Apr. 2014, doi: 10.1093/bioinformatics/btt656.

[17] R Core Team, “R: A language and environment for statistical computing. R Foundation for Statistical Computing, ienna, Austria https://www.R-project.org/,” 2020.

[18] M. I. Love, W. Huber, and S. Anders, “Moderated estimation of fold change and dispersion for RNA-seq data with DESeq2,” *Genome Biol*, vol. 15, no. 12, p. 550, Dec. 2014, doi: 10.1186/s13059-014-0550-8.

[19] Y. Zhou *et al.*, “Metascape provides a biologist-oriented resource for the analysis of systems-level datasets,” *Nat Commun*, vol. 10, no. 1, p. 1523, Dec. 2019, doi: 10.1038/s41467-019-09234-6.

[20] A. Janic, L. Mendizabal, S. Llamazares, D. Rossell, and C. Gonzalez, “Ectopic expression of germline genes drives malignant brain tumor growth in Drosophila,” *Science (1979)*, vol. 330, no. 6012, 2010, doi: 10.1126/science.1195481.

[21] C. Berger *et al.*, “FACS Purification and Transcriptome Analysis of Drosophila Neural Stem Cells Reveals a Role for Klumpfuss in Self-Renewal,” *Cell Rep*, 2012, doi: 10.1016/j.celrep.2012.07.008.

[22] D. Ershov *et al.*, “Bringing TrackMate into the era of machine-learning and deep-learning,” *bioRxiv*, p. 2021.09.03.458852, Jan. 2021, doi: 10.1101/2021.09.03.458852.
[23] U. Schmidt, M. Weigert, C. Broaddus, and G. Myers, “Cell Detection with Star-Convex Polygons,” 2018, pp. 265–273. doi: 10.1007/978-3-030-00934-2_30.
[24] J. Q. Boone and C. Q. Doe, “Identification of Drosophila type II neuroblast lineages containing transit amplifying ganglion mother cells,” *Dev Neurobiol*, 2008, doi: 10.1002/dneu.20648.
[25] S. K. Bowman, V. Rolland, J. Betschinger, K. A. Kinsey, G. Emery, and J. A. Knoblich, “The Tumor Suppressors Brat and Numb Regulate Transit-Amplifying Neuroblast Lineages in Drosophila,” *Dev Cell*, vol. 14, no. 4, pp. 535–546, Apr. 2008, doi: 10.1016/j.devcel.2008.03.004.
[26] B. C. Bello, N. Izergina, E. Caussinus, and H. Reichert, “Amplification of neural stem cell proliferation by intermediate progenitor cells in Drosophila brain development,” *Neural Dev*, vol. 3, no. 1, p. 5, 2008, doi: 10.1186/1749-8104-3-5.
[27] G. S. Marques, J. Teles-Reis, N. Konstantinides, P. H. Brito, and C. C. F. Homem, “Fate transitions in Drosophila neural lineages: a single cell road map to mature neurons,” *bioRxiv*, 2021, doi: 10.1101/2021.06.22.449317.
[28] K. Rust, M. D. Tiwari, V. K. Mishra, F. Grawe, and A. Wodarz, “Myc and the Tip60 chromatin remodeling complex control neuroblast maintenance and polarity in *Drosophila*,” *EMBO J*, vol. 37, no. 16, Aug. 2018, doi: 10.15252/embj.201798659.
[29] T. Ohshiro, T. Yagami, C. Zhang, and F. Matsuzaki, “Role of cortical tumour-suppressor proteins in asymmetric division of Drosophila neuroblast.,” *Nature*, vol. 408, no. 6812, pp. 593–6, Nov. 2000, doi: 10.1038/35046087.
[30] É. Kurucz *et al.*, “Nimrod, a Putative Phagocytosis Receptor with EGF Repeats in Drosophila Plasmatocytes,” *Current Biology*, vol. 17, no. 7, pp. 649–654, Apr. 2007, doi: 10.1016/j.cub.2007.02.041.
[31] E. Kurucz *et al.*, “Hemese, a hemocyte-specific transmembrane protein, affects the cellular immune response in *Drosophila*,” *Proceedings of the National Academy of Sciences*, vol. 100, no. 5, pp. 2622–2627, Mar. 2003, doi: 10.1073/pnas.0436940100.
[32] L. Horn, J. Leips, and M. Starz-Gaiano, “Phagocytic ability declines with age in adult <scp>D</scp> rosophila hemocytes,” *Aging Cell*, vol. 13, no. 4, pp. 719–728, Aug. 2014, doi: 10.1111/acel.12227.
[33] S. Chakrabarti and S. S. Visweswariah, “Intramacrophage ROS Primes the Innate Immune System via JAK/STAT and Toll Activation,” *Cell Rep*, vol. 33, no. 6, p. 108368, Nov. 2020, doi: 10.1016/j.celrep.2020.108368.
[34] A. Gyoergy *et al.*, “Tools Allowing Independent Visualization and Genetic Manipulation of *Drosophila melanogaster* Macrophages and Surrounding Tissues,” *G3 Genes|Genomes|Genetics*, vol. 8, no. 3, pp. 845–857, Mar. 2018, doi: 10.1534/g3.117.300452.
[35] U. Schmidt, M. Weigert, C. Broaddus, and G. Myers, “Cell Detection with Star-convex Polygons,” Jun. 2018, doi: 10.1007/978-3-030-00934-2_30.
